# Single-molecule chromatin tracing reveals a diversity of megabase heterochromatin domains

**DOI:** 10.1038/s41594-026-01895-4

**Published:** 2026-10-02

**Authors:** Dania Camila Pulido-Barrera, Jia E. You, Angelo L. Angonezi, Alexander Kuznetsov, Fei Xu, Tobias Verheijen, Ahilya N. Saw, Pavel Kos, Luca Giorgetti, Nacho Molina, David Brückner, Susan E. Mango

**Affiliations:** 1 Biozentrum, University of Basel, Basel, Switzerland; 2 Friedrich Miescher Institute for Biomedical Research (FMI), Basel, Switzerland; 3 Institute of Genetics, Molecular and Cellular Biology, Illkirch, France; 4 Department of Physics, University of Basel, Basel, Switzerland; 5 Present Address: Department of Biochemistry, University of Toronto, Toronto, Ontario Canada; 6 Present Address: IE University, Madrid, Spain

**Keywords:** Genome, Epigenomics, Chromatin, Chromatin structure, Chromosomes

## Abstract

Chromosomes fold into distinct domains that regulate transcription, replication and repair. In addition to well-characterized topologically associating domains (TADs) and compartments, the diversity of heterochromatin domains remains poorly defined at the sequence level. Here, using single-molecule tracing of nascent heterochromatin in *Caenorhabditis* *elegans*, we identify three classes of megabase-scale domains: (1) sharp-boundary, condensin I-dependent TAD-like domains (TADLs), which arise before zygotic genome activation; (2) similarly sized, but condensin-independent, *elegans* condensin-independent domains (elCIDs), one of which encompasses the 5S-spliced leader repeat locus; and (3) decompacted, diffuse structures that are abundant in the population but probably missed by crosslinking assays. Condensin I sits at the border of the TADL domain, whereas DNA-binding proteins, active histone marks and maternally active genes flank the elCID domains, suggesting that boundaries are established by distinct mechanisms for different types of domains. Our results uncover previously unrecognized heterochromatin architectures and implicate maternal transcriptional activity in shaping domain boundaries in early embryos in *C.* *elegans*.

## Abstract (web summary)

Single-molecule imaging reveals diverse, megabase-scale heterochromatin domains in *C.* *elegans*. Some resemble TADs and depend on condensin I; others are independent of condensin and histone H3K9me, including one domain that encloses the 5S rDNA locus.

## Main

Recent advances in chromatin biology show that the genome is organized into domains across multiple scales. At the megabase scale, Hi-C revealed TADs, while histone modifications drive segregation into euchromatic and heterochromatic compartments^1–4^, shaping gene regulation, genome integrity and nuclear organization^5–9^. Microscopy-based approaches have revealed additional structures—packing domains, Polycomb bodies and the nucleolus—reflecting chromatin density at diverse scales^10–15^. Packing domains, for instance, do not always coincide with Hi-C-defined TADs or compartments, suggesting complementary modes of genome folding that elude crosslinking-based approaches.

Early models proposed a hierarchical architecture of multi-megabase A/B compartments subdivided into TADs^3,16,17^. High-resolution 3C-based maps now reveal a more dynamic, multilayered configuration, with CTCF-anchored loops, compartments and finer-scale features coexisting^17–19^, favoring mechanistic over size-based or contact-based classifications. In higher organisms, loop extrusion by structural maintenance of chromosomes (SMC) complexes shapes chromatin domains: cohesin generates transient loops within TADs^7,20–26^, whereas condensin builds interphase domains in vertebrate cells and *C.* *elegans*^27–29^.

In *C.* *elegans*, megabase-scale TADs occur on the X chromosome, where a specialized condensin I complex (condensin I^DC^) generates both TADs and their boundaries^22,30^. Condensin I also shapes autosomal organization, although clear megabase-scale TADs have not been observed^22,23,31^. Instead, autosomes feature small, cohesin-dependent TADLs spanning 20–40 kb^32^. This raises the question of whether larger chromatin domains also exist in *C.* *elegans*, like those in vertebrates.

Here, we explore the organization of autosomes at the megabase scale, focusing on a stretch of heterochromatin that undergoes large-scale folding^33^. Using chromatin tracing to achieve single-chromosome resolution^33–35^, we discover a diversity of chromatin domains with distinct genetic requirements and developmental timing.

## Results

### Single-molecule tracing defines well-insulated chromosome domains

**Fig. 1:**
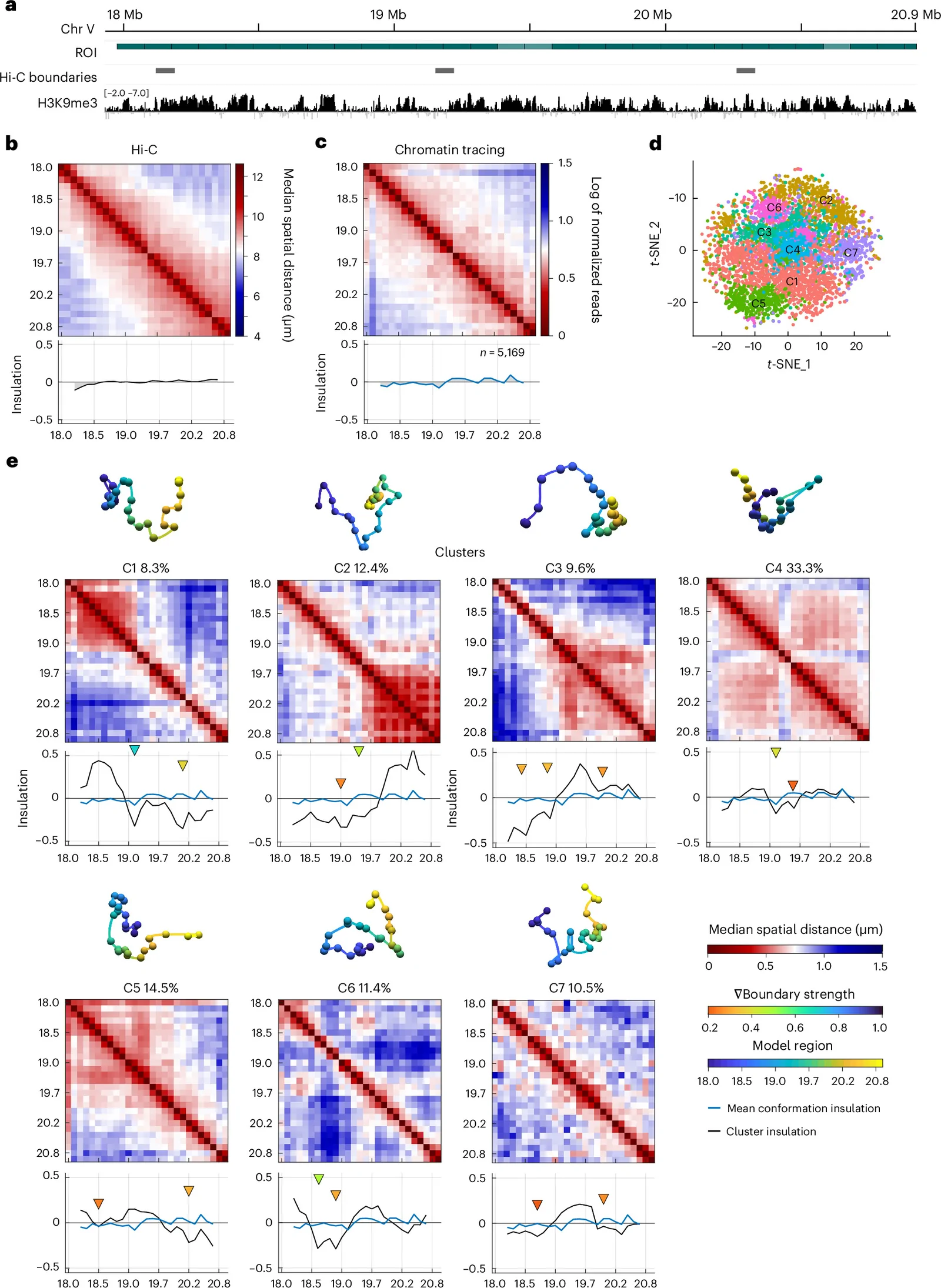
Single-molecule tracing reveals well-insulated chromosome domains in *C.* *elegans* embryos. **a**, Schematic of the chromosome V ROI traced in this study (dark green); untraced flanking regions are shown in light green. Gray ticks mark Hi-C-defined TAD boundaries^22^, and the H3K9me3 ChIP–seq signal is plotted below^76^. **b**, The log of normalized Hi-C sequencing read counts at 100 Kb resolution across the ROI with insulation score^31^. **c**, Median pairwise distance matrix (μm) for chromosomes at developmental stages spanning 2–140 cells with insulation scores. **d**, *t*-distributed stochastic neighbor embedding (*t*-SNE) plot of Chr V clusters; each dot represents a single chromosome trace and each color denotes a cluster. **e**, Median pairwise distance matrices (μm) for each cluster identified in **d**, with insulation scores shown below. Triangles mark TAD boundary positions, colored by boundary strength. Above each matrix, three-dimensional bead-on-a-string models reconstructed by classical multidimensional scaling illustrate cluster-averaged conformations; each sphere represents a genomic region positioned according to its median pairwise distances within the cluster.

**Extended Data Fig. 1:**
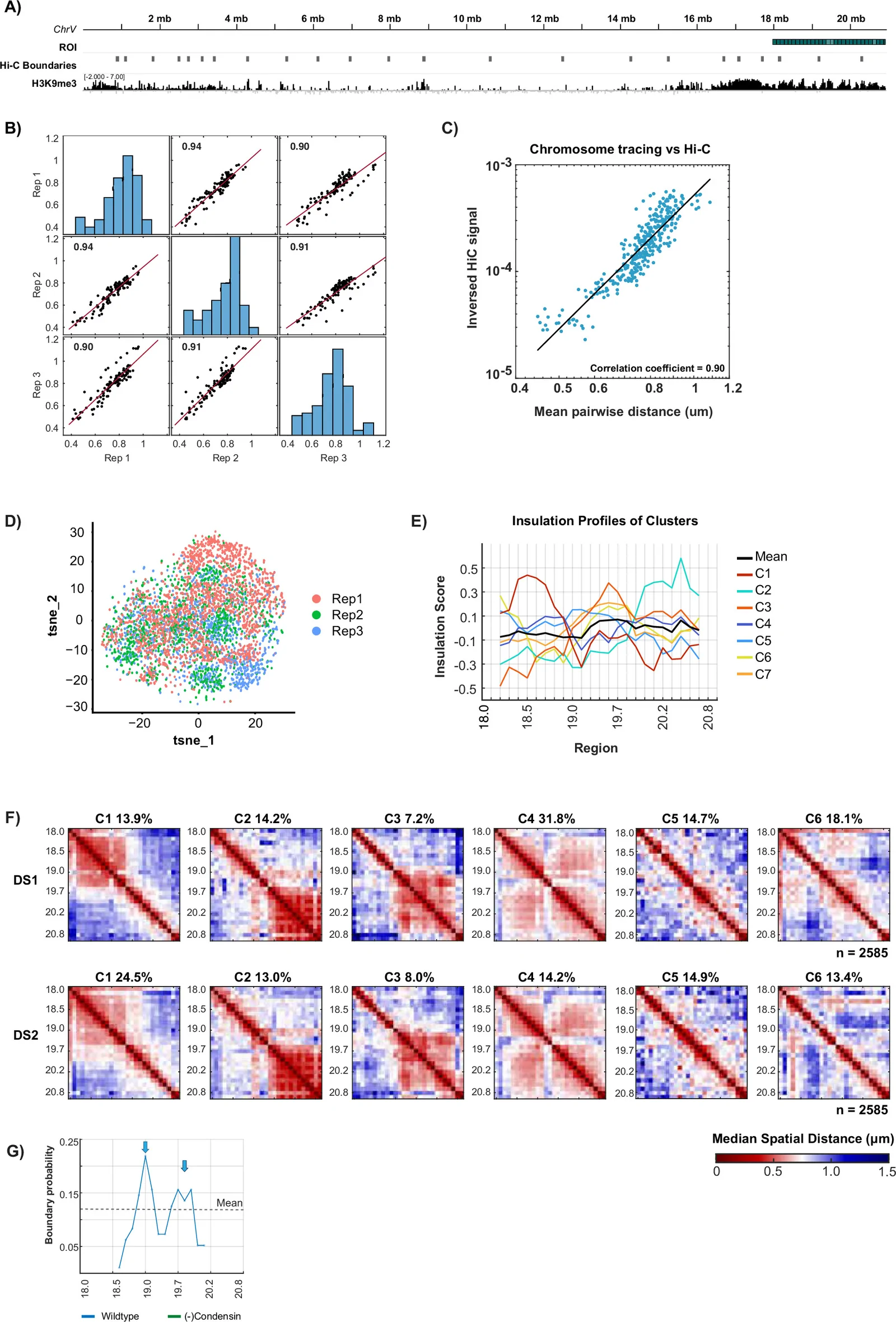
Single-molecule tracing replicates show high correlation among themselves and with hi-c data. **A**) Schematic of the Chromosome V. Region of interest (ROI) traced in this study (dark green). Gray ticks mark Hi-C–defined TAD boundaries^23^, and the H3K9me3 ChIP–seq signal is plotted below^79^. **B**) Pearson correlation between replicates for wild-type chromatin tracing experiments. **C**) Pearson correlation between median distances obtained from single-molecule tracing and inverse contact frequencies (1/Hi-C contact frequency) for the same region of interest. **D**) *t*-SNE representation showing the distribution of traces across biological replicates. **E**) Insulation profiles for all clusters. **F**) Two independent random downsamplings of traces to 50% (DS1 and DS2), followed by clustering as in the main analysis (Fig. 1e), showing that cluster identities are reproducible. **G**) Boundary probability for wild-type traces. Blue arrows indicate regions of high probability.

We reasoned that this apparent lack of well-defined domains could arise from population averaging across heterogeneous chromatin conformations, including those linked to cell cycle, cell type or development. To address this issue, we used chromatin tracing (100 kb probe spacing; Fig. 1a and Extended Data Fig. 1a,b), a DNA fluorescence in situ hybridization (FISH)-based technique, to determine chromatin conformations at the single-molecule level^33,34,36^. We excluded mitotic nuclei, yielding a dataset composed predominantly of interphase nuclei. Population-averaged tracing data closely resembled published Hi-C maps (*n* = 5,169 traces, *r* = 0.90; Extended Data Fig. 1c) and, like Hi-C, lacked clear boundaries or strong domain structures (Fig. 1b,c)^22,23,31,32^.

We performed unsupervised clustering of single-molecule traces (Methods), revealing groups with similar conformations^33,36^. In parallel, we quantified insulation and boundary strength using a previously published sliding window approach^22^. This analysis identified seven distinct conformational clusters (Fig. 1d,e). Three clusters exhibited ~1 Mb domains with sharp boundaries and strong insulation scores (0.5 to −0.4), nearly fourfold higher than the population average (Fig. 1e (clusters C1–C3) and Extended Data Fig. 1e). Clusters C1 and C2 showed the strongest boundaries and together accounted for ~20% of traces; these domains did not coexist within the same trace, instead appearing in an either/or configuration or interacting to form larger domains resembling cluster C4. The remaining clusters displayed weaker but above-average insulation values and distinct boundary positions, underscoring structural variability.

Single-molecule traces from three replicates showed strong *t*-distributed stochastic neighbor embedding overlap, confirming reproducibility (Extended Data Fig. 1d), and downsampled (50%) subsets yielded clustering similar to the full dataset (Extended Data Fig. 1f). Boundary position alternated between ~19.0 and ~19.7 Mb, enriched at both but preferentially at 19.0 (Extended Data Fig. 1g), indicating meaningful biological heterogeneity rather than sampling noise. We conclude that *C.* *elegans* chromosomes adopt diverse megabase-scale conformations, including a subset with sharply insulated domains; this heterogeneity probably explains the absence of clear domains in population-averaged maps and supports a model of dynamically regulated chromosome organization, consistent with recent live-imaging studies^21,37^.

### Condensin I is required for a *C.elegans* TAD-like heterochromatin domain

**Extended Data Fig. 2:**
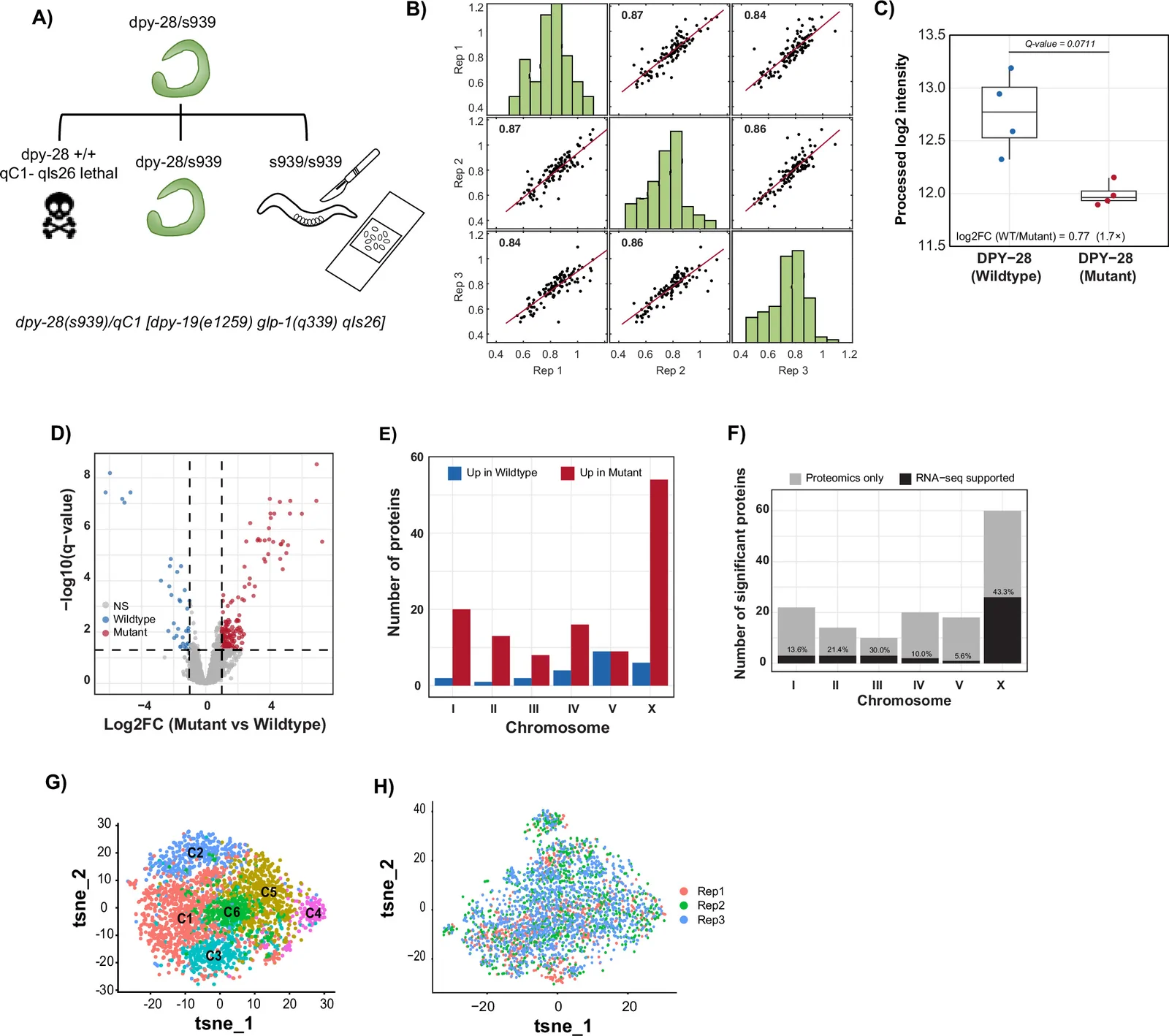
Condensin I loss affects chromosome V pairwise distances. **A**) Schematic representation of the experiment using the dpy-28(s939) mutant. **B**) Pearson correlation between replicates for condensin(-) chromatin tracing experiments**. C**) Processed log_2_ intensity values derived from label-free LC–MS/MS quantification for DPY-28 in wildtype and mutant samples. Points indicate individual measurements. The reported q-value and log_2_ fold change (WT/Mutant) indicate reduced abundance in the condensin(-) mutant. Box plots indicate the median (center line) and 25th and 75th percentiles (box bounds). **D**) Volcano plot showing differential protein abundance between condensin(-) and wildtype. The x-axis shows log_2_ fold change (condensin(-) vs Wildtype) and the y-axis shows −log_10_(q-value). Proteins meeting significance thresholds (q < 0.05 and |log_2_FC | ≥ 1) are highlighted, with increased abundance in mutant (red) and wildtype (blue); non-significant proteins are shown in gray. **E**) Chromosomal distribution of significantly changing proteins. Bars indicate the number of proteins upregulated in wildtype (blue) or condensin (-) (red) across each *C. elegans* chromosome. **F**) Stacked bars show the total number of proteomics genes per chromosome, subdivided into proteins detected only by proteomics (gray) or also supported by RNA-seq (red). Porcentage are the proprocion of traces detected in RNA-seq and proteomics **G**) t-SNE plot of Chromosome V (chrV) clusters in condensin(-) mutants, where each dot represents a single chromosome trace, and each color corresponds to a cluster. **H**) t-SNE representation showing the distribution of traces across biological replicates.

Megabase-scale genomic domains are often grouped into loop-extrusion-associated domains (for example, TADs) versus compartments arising from preferential interactions between chromatin regions with similar epigenetic states^2,4,8,19^. In *C.* *elegans*, cohesin forms small loops (~20–40 kb)^38,39^, whereas condensin I folds the genome at a larger scale^22,23^. Given the megabase size of our domains, we focused on *dpy-28* (Extended Data Fig. 2a,b), which encodes a condensin I subunit^40,41^. *dpy-28(s939)* is a strong loss-of-function allele impairing condensin I activity and dosage compensation^40,42^, with DPY-28 undetectable by antibody-based assays in mutant embryos^40^. Label-free liquid chromatography–tandem mass spectrometry (LC–MS/MS) analysis showed that DPY-28 remained detectable but at reduced levels in the mutant (Extended Data Fig. 2c and Supplementary Table 1). Consistent with condensin’s role in dosage compensation^22,23,41,43,44^, the most significantly changed proteins were X-linked (Extended Data Fig. 2d–f) and overlapped substantially with genes upregulated upon perturbation of the condensin I subunit DPY-26 (ref. ^23^). Condensin II remains intact in dpy-28 mutants, probably explaining their ability to undergo mitosis and survive to larval stages^45^.

**Fig. 2:**
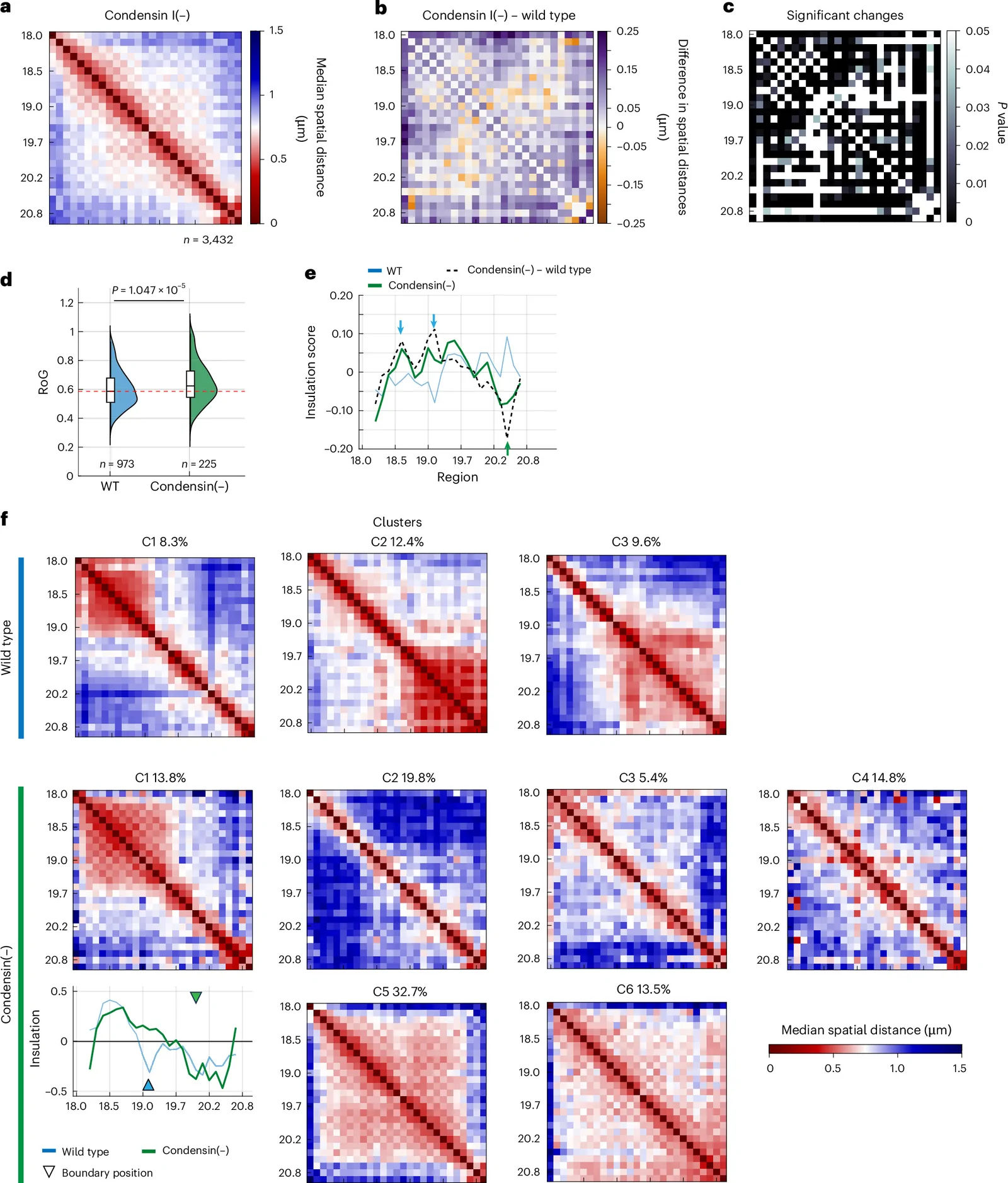
Condensin I is required for a subset of *C.* *elegans* heterochromatin domains. **a**, Median pairwise distance matrix (μm) for condensin mutant from 2 to 140 cells, annotated with insulation scores. Model of condensin I to the right. **b**, Difference in pairwise distances (μm) between condensin(−) mutants and wild-type traces. Purple regions indicate increased distances in condensin mutants, while orange regions indicate increased distances in wild type. **c**, Significant changes in pairwise distances between wild type and condensin(−) mutants, determined by one-tailed Welch’s *t*-test. **d**, RoG for wild-type (WT) and condensin(−) traces. Statistical significance was determined using a two-tailed *t*-test. Boxplots indicate the median (center line) and 25th and 75th percentiles (box bounds); violin outlines show the full distribution of the data. *n* indicates the number of traces. Dashed red line indicates the wild-type median. **e**, Insulation scores for wild-type, condensin(−) average matrices and their difference. Blue arrows highlight regions with reduced insulation after condensin depletion, while green arrows indicate regions with increased insulation. **f**, Median pairwise distance matrices (μm) for each cluster. The top row displays clusters with distinct domain boundaries in wild-type, while the bottom row shows clusters in condensin(−) mutants. The insulation scores display the insulation of both C1 clusters in wild-type and condensin(−) embryos. Green arrows mark boundaries in condensin mutants; blue arrows mark boundaries in wild-type.

At the population level, median distance matrices from three replicates revealed that condensin-deficient embryos displayed an alternating, checkered pattern of longer and shorter distances rather than the continuous domain-like interactions seen in wild type (*n* = 3432 traces; Fig. 2a–c), with nearly all probe pairs showing significantly increased distances, indicating reduced compaction (Fig. 2b,c). Radius of gyration (RoG) measurements confirmed significantly larger chromosomal volumes in *dpy-28* mutants relative to wild type (*P* = 1.05 × 10^−^^5^; Fig. 2d), consistent with global decompaction. Two regions (18.6 and 19.1 Mb) showed increased proximity, indicating a loss of insulation (Fig. 2e), while a poorly insulated region in wild type (20.4 Mb) became more insulated. These data suggest global chromatin restructuring upon condensin I loss.

We performed clustering using the same parameters applied to the wild-type dataset (Fig. 2f and Extended Data Fig. 2g,h). We focused on clusters displaying the strongest boundaries in the wild type (Fig. 2f, top row). Two prominent wild-type domains (WT-C1, WT-C2) were differentially affected in *dpy-28* mutants: the distal domain (19.7–20.9 Mb; C2) was lost, and based on this condensin dependence, sharp boundaries and size, we refer to it as a TADL. By contrast, the proximal domain (18.2–19.6 Mb; C1) persisted, although slightly decompacted, with weaker boundaries; we refer to this cluster as elCID, which was more abundant in condensin mutants than in the wild type (~14% vs ~8%). The remaining traces resolved into either highly open (clusters C2–C4; ~40%) or compact conformations spanning the region (clusters C5–C6; ~46%) (Fig. 2f, bottom rows).

**Extended Data Fig. 3:**
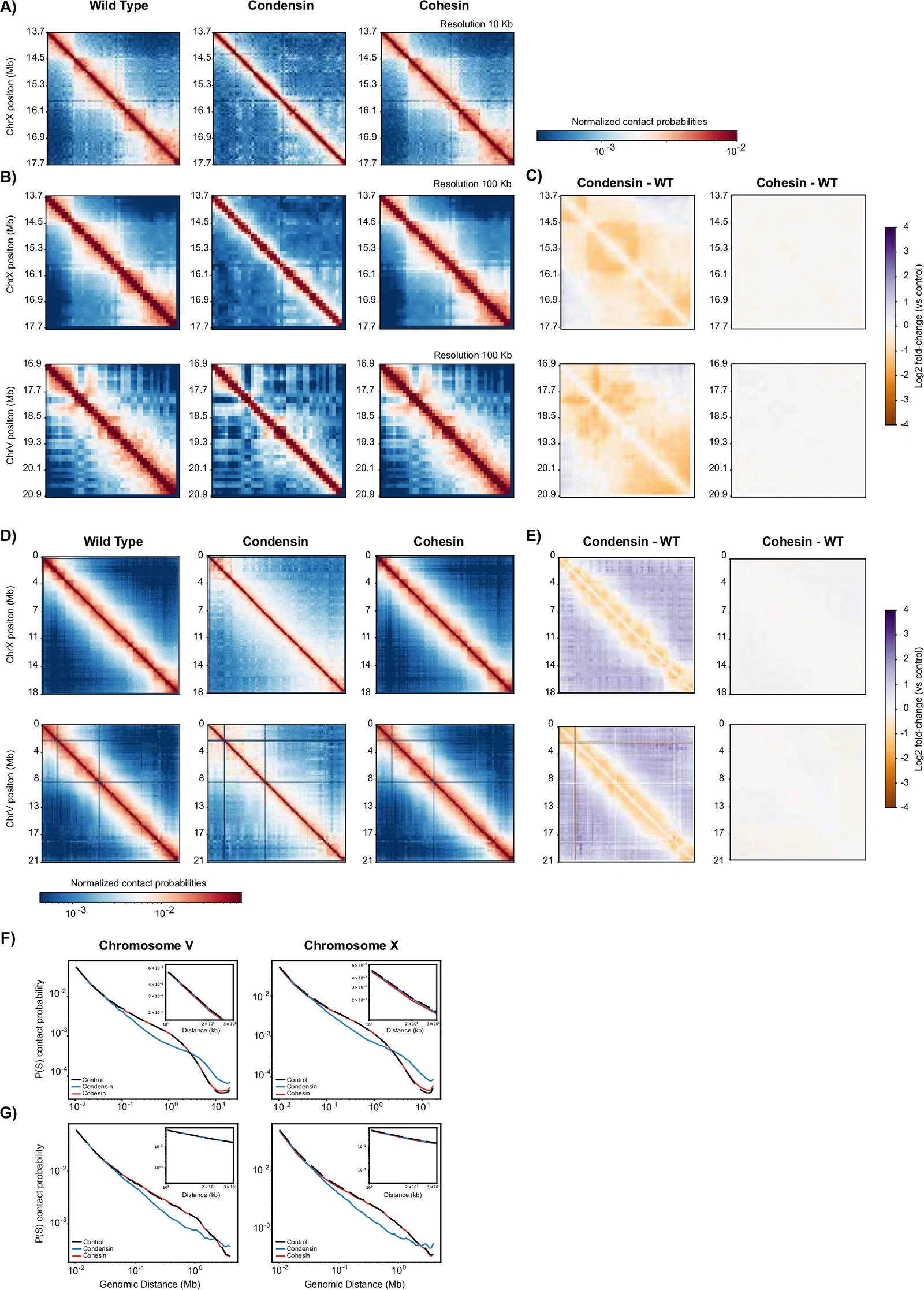
Structural alterations at 100 kb resolution are driven by condensin depletion, not Cohesin loss. **A-B**) Hi-C contact maps for wild type, condensin-depleted, and Cohesin-depleted embryos shown at 10 kb (**A**) and 100 kb (**B**) resolution^24^. Cohesin-dependent local features are visible at 10 kb resolution but are largely averaged out at 100 kb resolution, matching the resolution of the chromatin tracing experiments. **C**) Log_2_ fold-change contact maps (condensin − WT and Cohesin − WT). **D-E**) Whole chromosome showing contact maps and corresponding log_2_ fold-change maps (**E**) at 100 kb resolution. Structural alterations are consistently observed upon Condensin depletion but are largely absent following Cohesin depletion. **F-G**) Contact probability scaling plots, P(s), for chromosomes V (**F**) and X (**G**). Condensin depletion leads to a steeper decay of P(s), indicative of reduced intermediate- and long-range interactions, whereas Cohesin depletion produces curves that closely overlap with wild type.

Given that elCID persisted upon condensin I depletion, we tested whether cohesin also contributed to chromatin organization at this scale. Cohesin-dependent loops are detectable in Hi-C at ~10 kb resolution^38,39^ but were averaged out at the 100 kb resolution used here (Extended Data Fig. 3a,b). The log_2_(fold change) contact maps showed pronounced alterations upon condensin depletion but minimal changes upon cohesin depletion across multiple chromosomes (Extended Data Fig. 3c–e). Contact probability scaling further supported this distinction: condensin depletion produced a steeper decay, indicating loss of intermediate and long-range contacts, whereas cohesin depletion yielded curves nearly indistinguishable from wild type (Extended Data Fig. 3f,g). Together, these analyses indicate that cohesin organizes chromatin at smaller scales but not at megabase resolution.

### Histone H3K9 methylation mediates compaction but not boundary formation

**Fig. 3:**
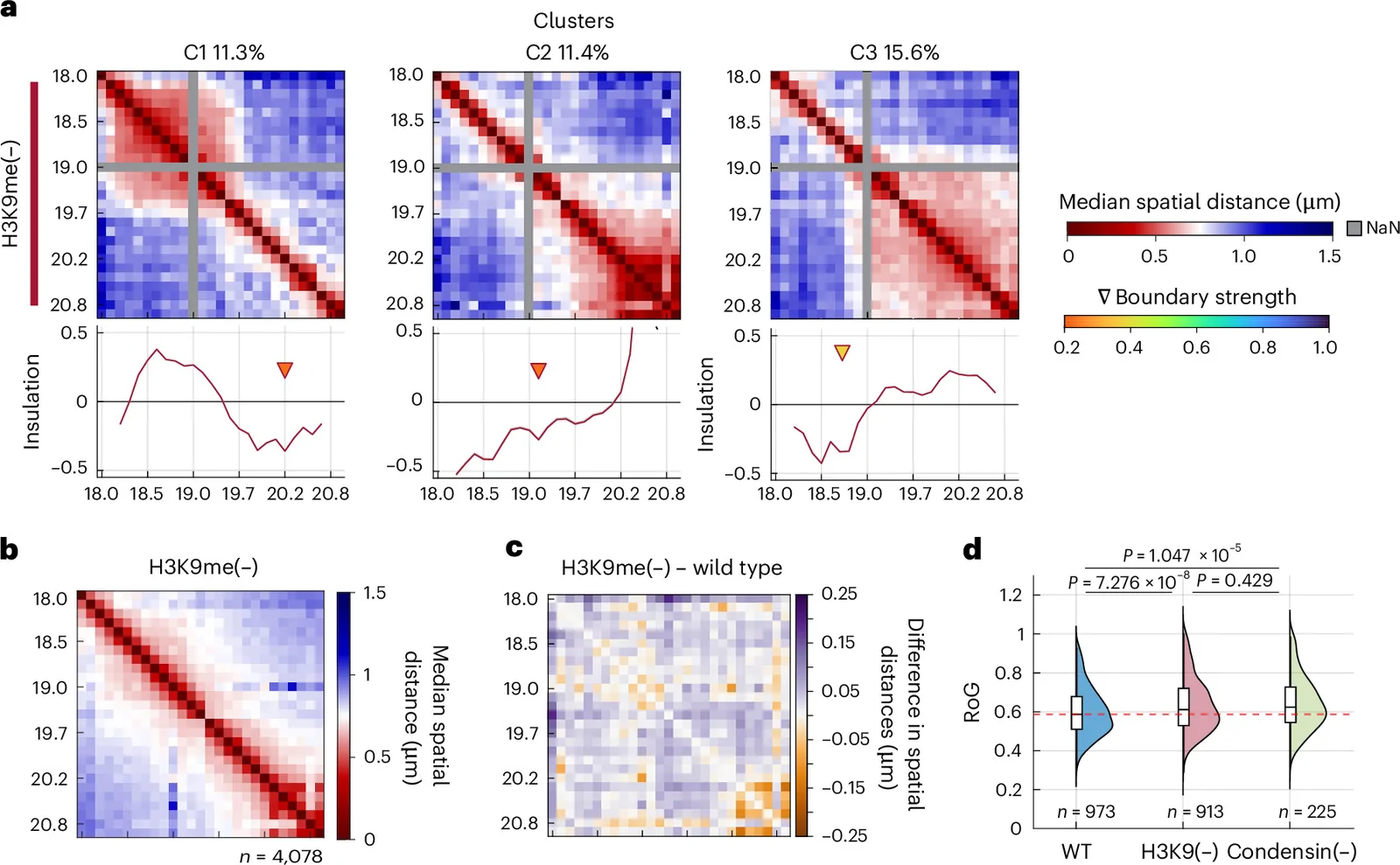
H3K9me mediates compaction but not boundary formation of heterochromatin domains. **a**, Median pairwise distance matrices (μm) for C1, C2 and C3 clusters in H3K9me traces, annotated with insulation scores. Triangles indicate TAD boundary positions, with colors representing boundary strength. NaN (gray) indicates regions with missing values. **b**, Median pairwise distance matrix (μm) for H3K9me(−) mutant chromosomes across developmental stages from 2 to 140 cells. **c**, Differences in pairwise distances between H3K9me(−) mutants and wild-type traces. Purple regions indicate distances larger in H3K9me(−) mutants, while orange regions indicate distances larger in wild type. **d**, RoG for wild-type, H3K9me(−) and condensin(−) traces. Statistical significance was determined using a two-tailed *t*-test. Boxplots indicate the median (center line) and 25th and 75th percentiles (box bounds); violin outlines show the full distribution of the data. *n* indicates the number of traces. Dashed red line indicates the wild-type median.

**Extended Data Fig. 4:**
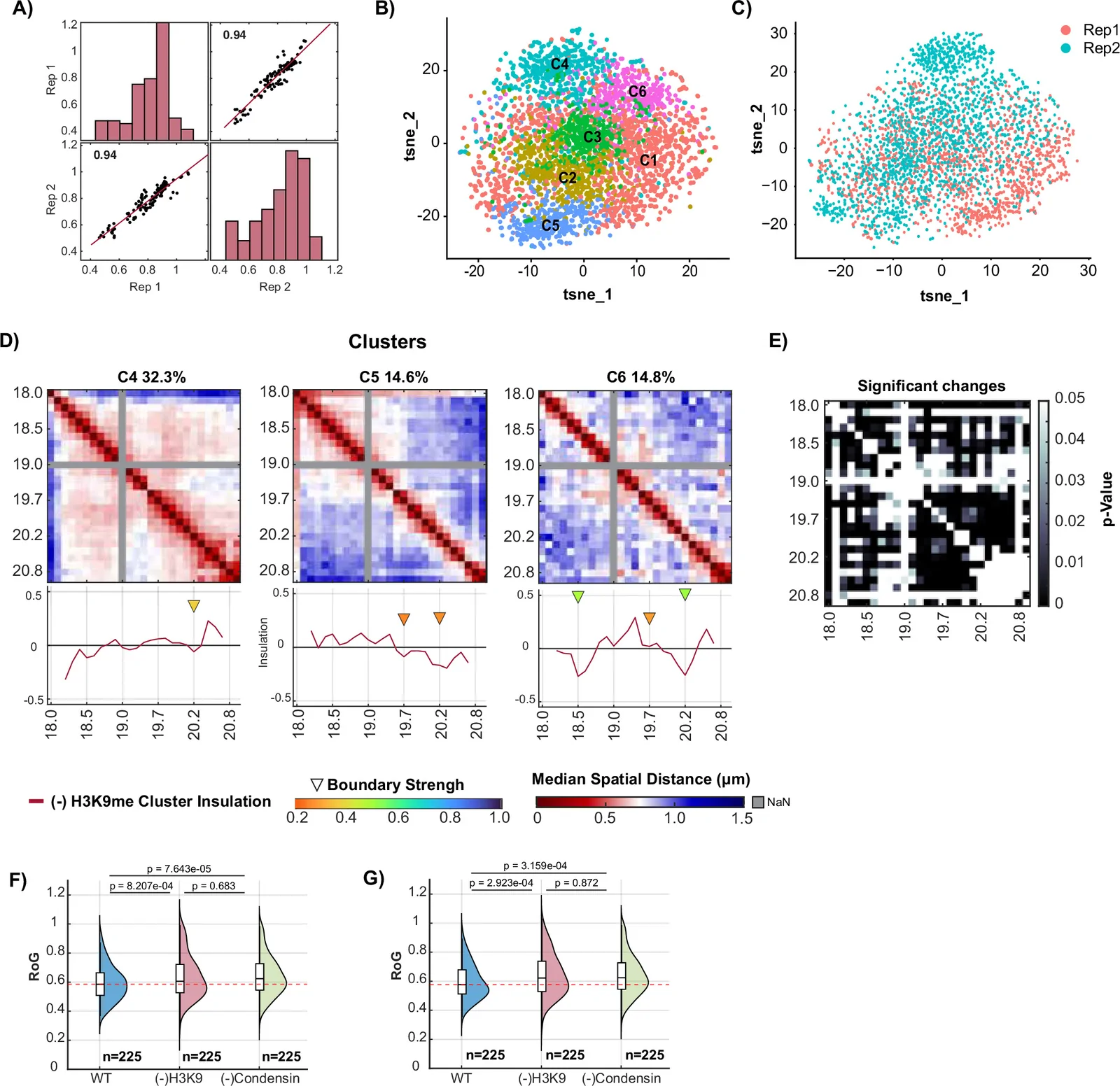
Pairwise distance, clustering, and structural changes in H3K9me(-) mutants. **A**) Pearson correlation between replicates for wild-type chromatin tracing experiments, demonstrating data reproducibility. **B**) t-SNE plot for clusters in H3K9me(-) mutants, where each dot represents a single chromosome trace and each color corresponds to a cluster. **C**) *t*-SNE representation showing the distribution of traces across biological replicates. **D**) Median pairwise distance matrices (μm) for C4, C5, and C6 clusters in H3K9me traces, annotated with insulation scores. Triangles mark TAD boundary positions, with colors indicating boundary strength. NaN (gray) indicates regions with missing values. **E**) Significant changes in pairwise distances between wild-type and H3K9me(-) mutants, determined by one-tailed Welch’s t-test. **F-G**) Radius of gyration for wild-type, H3K9me(-), and condensin(-) traces, down sampled to ensure an equal number of measurements across conditions. Statistical significance was determined using a two-tailed t-test. Box plots indicate the median (center line) and 25th and 75th percentiles (box bounds); violin outlines show the full distribution of the data. n indicates the number of traces. Dashed red line indicates the wild-type median.

Epigenetic modifications contribute to compartmentalization, whereas loop extrusion factors act largely independently of chromatin state^1,3,18,46–48^. If C2 corresponds to a TADL domain, its formation should not require H3K9 methylation. We analyzed embryos lacking virtually all H3K9 methylation (*met-2;* *set-25* double mutants)^49,50^. Clustering of two independent replicates, using the same parameters as for wild type (Fig. 3a and Extended Data Fig. 4a–d), identified domain-containing clusters similar to those in wild type (Fig. 1e), including both the elCID and the TADL domain (Fig. 3a), indicating that these megabase-scale domains form independently of H3K9 methylation.

H3K9 methylation compacts chromosomes during large-scale heterochromatin formation^46,50,51^. We measured median distances and RoG in wild type and *met-2;* *set-25* mutant embryos, which revealed increased distances across the region in mutant embryos, indicating reduced compaction (*n* = 4078 traces; Fig. 3b,c and Extended Data Fig. 4e). RoG analysis confirmed significantly larger chromosome volumes in mutants (*P* = 7.28 × 10^−^^8^), comparable to *dpy-28* embryos (*P* = 0.429) (Fig. 3d), and downsampling controls ruled out sample-size bias (Extended Data Fig. 4f,g). These results show that H3K9 methylation is required for global chromatin compaction, affecting both condensin-dependent and independent domains, but is not required for either elCID or the TADL per se.

### Distinct genetic dependencies shape TADL and elCID chromatin structures

**Fig. 4:**
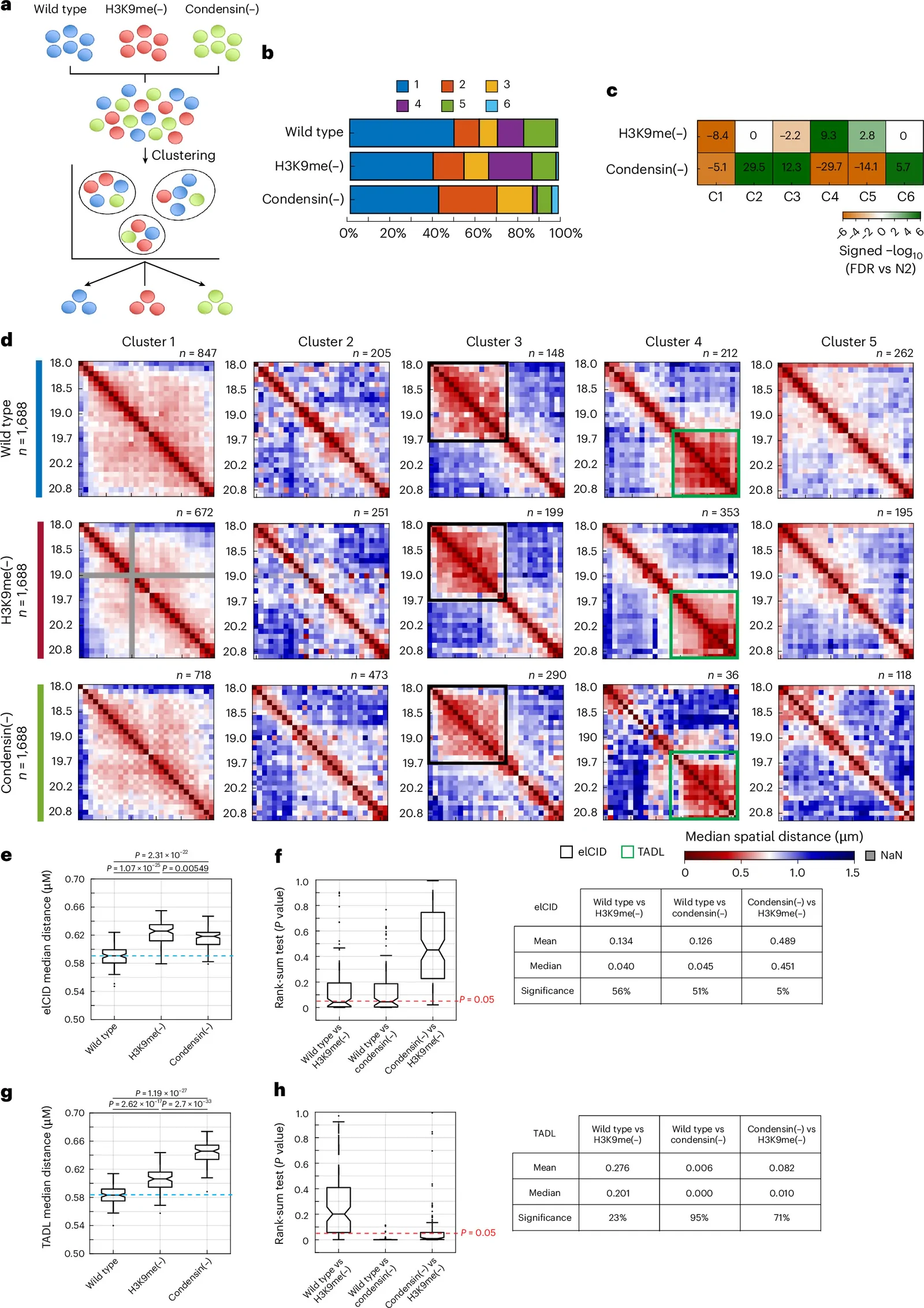
Chromosomal clustering highlights condensin’s essential role in TAD-like organization. **a**, Schematic representation of the co-clustering strategy used to group chromosome traces based on structural similarities. **b**, Contribution of each genotype to the clusters identified using the strategy outlined in **a**, illustrating how different genotypes are distributed across structural clusters. **c**, Heatmap of signed −log_10_(FDR) enrichment scores across clusters, with green indicating enrichment and orange depletion relative to wild type. **d**, Median pairwise distance matrices (μm) for each identified cluster, shown separately by genotype. Co-clustering was performed by pooling chromosome traces from different genotypes. NaN (gray) indicates regions with missing values. **e**–**h**, Iterative downsampling analysis of elCID (**e**,**f**) and TADL (**g**,**h**) spatial distances across wild-type, H3K9me(−) and condensin(−) conditions. Boxplots summarize median distances obtained from 100 independent downsampling iterations (1,000 distances per iteration). Boxplots in **e** and **g** show median spatial distances per condition; box bounds indicate the 25th and 75th percentiles, the center line indicates the median and whiskers extend to the most extreme value within 1.5× the interquartile range; horizontal bars mark medians, and *P* values from full datasets are shown above. Panels **f** and **j** show corresponding boxplots of *P* values from pairwise two-sided Wilcoxon rank-sum tests across the same iterations, defined as in **e** and **g**, with the red dashed line indicating *P* = 0.05. Adjacent tables report mean and median *P* values and the fraction of iterations with *P* < 0.05, reflecting the robustness of differences observed in **e** and **g**.

**Extended Data Fig. 5:**
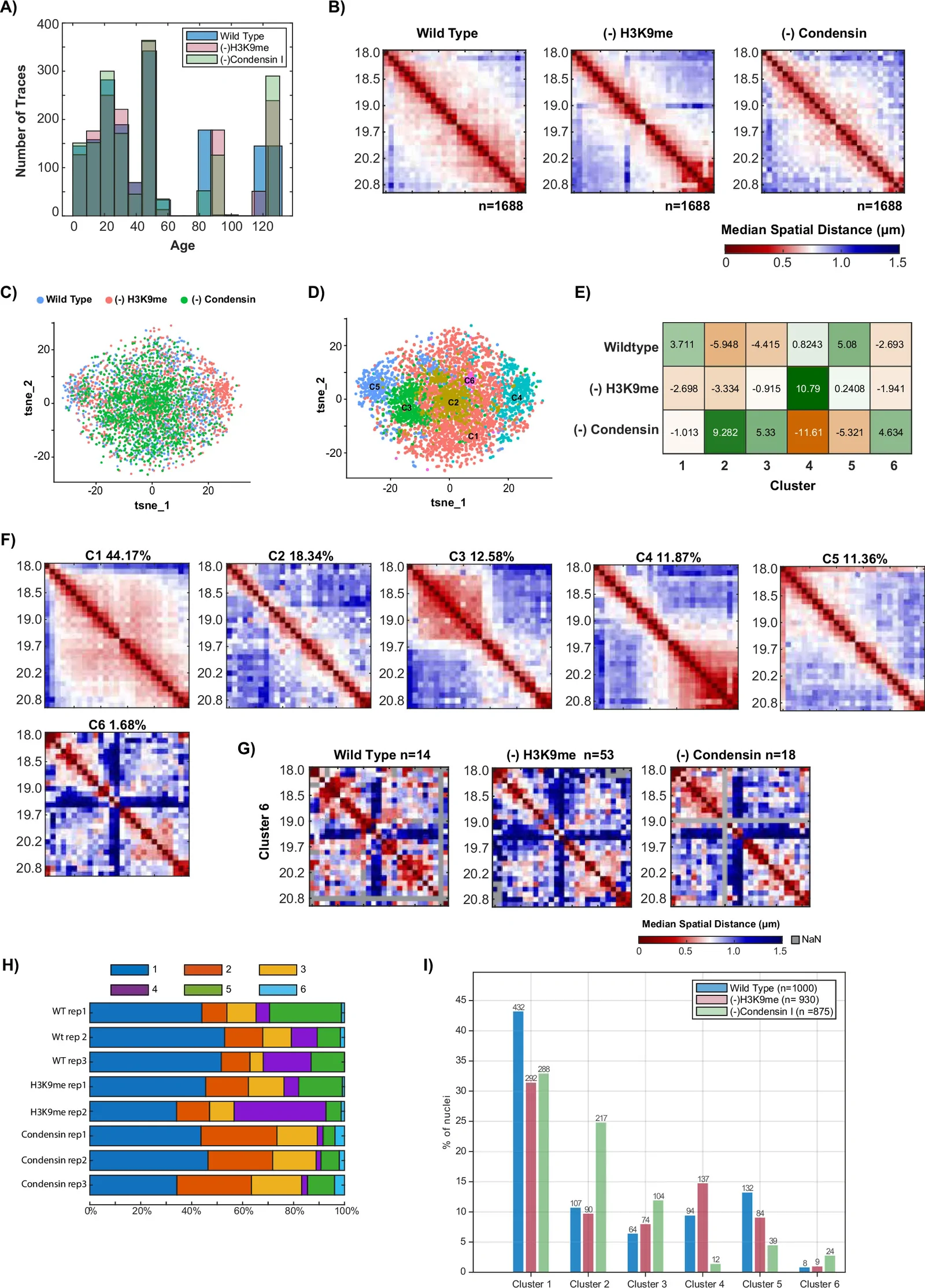
Median matrices after downsampling show no changes in interaction patterns. **A**) Age distribution of the downsampled dataset used in Fig. 4 (see Methods for details). **B**) Median pairwise distance matrices (μm) after downsampling to match the distribution in (A). **C**) t-SNE plot of the co-clustering analysis (as in Fig. 4a), with each dot representing a single chromosome trace and colors denoting genotypes. **D**) t-SNE plot of clusters from the co-clustering analysis; dots represent single traces, colored by cluster identity. **E**) Standardized chi-square residuals from the co-clustering analysis in Fig. 4d. High positive values indicate enrichment of traces from a genotype within a cluster, while negative values indicate depletion. **F**) Median pairwise distance matrices (μm) for the clusters in (D), before splitting by genotype. **G**) Median pairwise distance matrices (μm) for cluster 6. n indicates the number of traces. **H**) Bar plot showing the distribution of biological replicates for each genotype across clusters. **I**) Percentage of nuclei in each structural cluster (C1–C6) identified from pooled co-clustering (Fig. 4a–d), shown separately for wild-type (n = 1000), H3K9me(–) (n = 930), and condensin(–) (n = 875) traces. Numbers above bars indicate absolute trace counts per cluster.

Clustering traces separately by genotype prevents direct comparison of structurally similar clusters across conditions (for example, C1 in wild type is not equivalent to C1 in H3K9me mutants). We therefore pooled and clustered traces from wild-type and mutant embryos (Fig. 4a), downsampling to match age distributions across genotypes (1,688 traces per genotype; Extended Data Fig. 5a,b and Methods), enabling unbiased comparison of shared and genotype-specific conformations and statistical testing of any differences.

Clustering the pooled traces revealed genotype-specific differences in chromatin domain distribution (Fig. 4b–d and Extended Data Fig. 5c–h), with cluster composition differing significantly between genotypes (χ^2^_10_ = 541.95, *P*  = 4.7 × 10^−^^110^; Cramér’s *V* = 0.23). Standardized residual analysis identified strong enrichment or depletion of specific clusters within individual genotypes, indicating that distinct folding states were preferentially associated with different genetic backgrounds (Extended Data Fig. 5e and Supplementary Table 2), visualized as signed −log_10_(false discovery rate (FDR)) enrichment scores relative to wild type (Methods and Fig. 4c). Observed and expected counts, standardized residuals and FDR-adjusted *P* values for all genotype–cluster combinations are provided in Supplementary Table 2.

The most pronounced difference was cluster C4, the TADL domain: present in wild type, enriched in H3K9me mutants but nearly absent in dpy-28 mutants (χ^2^ residual = –11.61, FDR < 10⁻^6^). Cluster C3, the elCID (18.2–19.6 Mb), was enriched in condensin mutants (χ^2^ residual = 5.3) but unaffected by H3K9me loss, highlighting that structurally similar domains can arise through distinct regulatory factors. Cluster C2, a more decompacted conformation, was significantly enriched in dpy-28 mutants (χ^2^ residual = 9.3) and roughly half as frequent in wild type and H3K9me mutants (Fig. 4d and Extended Data Fig. 5e).

**Extended Data Fig. 6:**
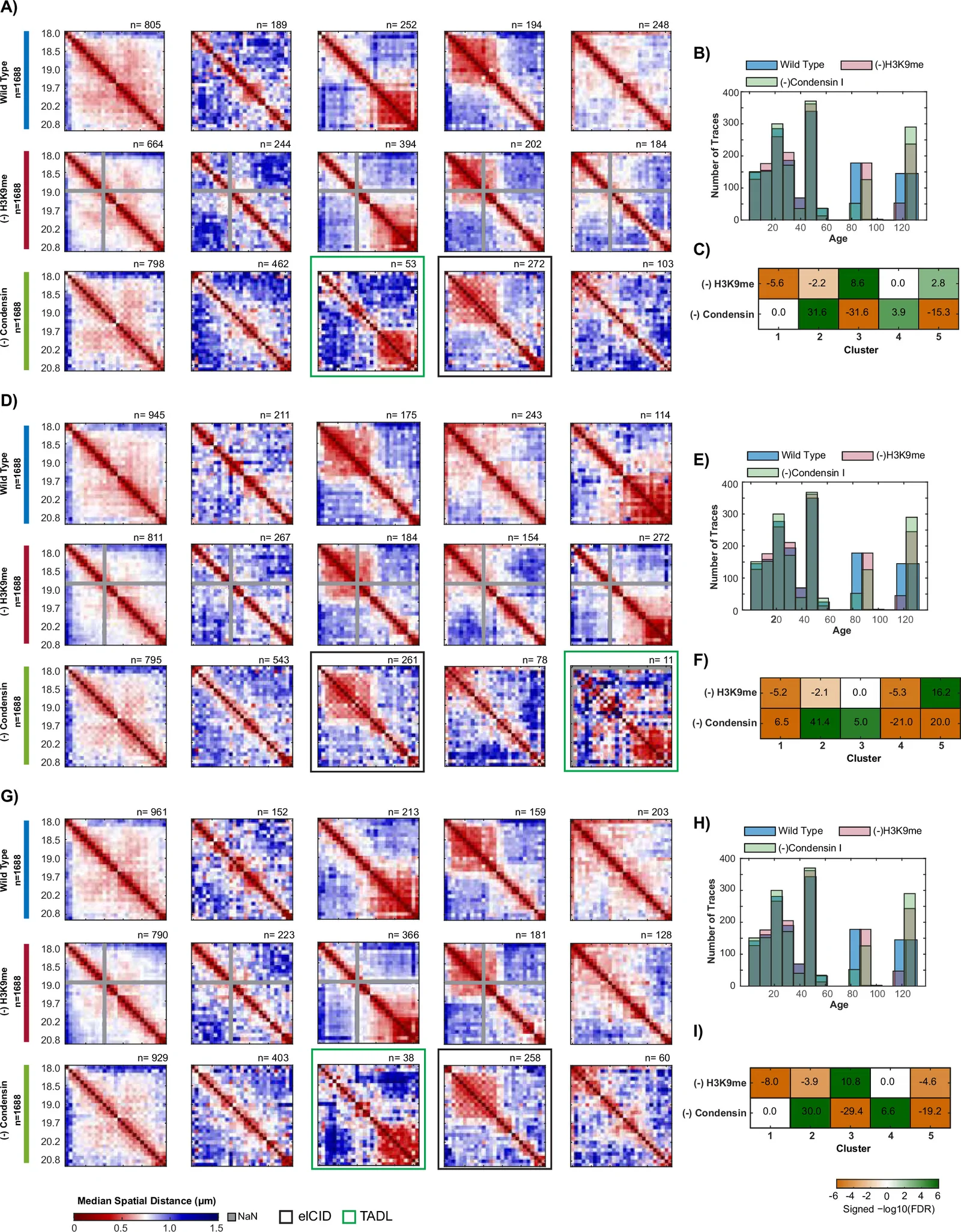
Chromatin domain organization is preserved upon downsampling. Independent co-clustering using downsampled data following the same age distribution as in Fig. 4. **A,D,G**) Median pairwise distance matrices (μm) for each identified cluster, shown separately by genotype. Co-clustering was performed by pooling chromosome traces from different genotypes. NaN (grey) indicates regions with missing values. Boxes outline clusters corresponding to elCID or TALD domains. n indicates the number of traces. **B,E,H**) Age distribution of traces in each downsample used for clustering, corresponding to A, D and G, respectively. **C,F,I**) Heatmap of signed −log_10_(FDR) enrichment scores across clusters, with green indicating enrichment and orange indicating depletion relative to wild type.

We calculated the fraction of nuclei assigned to each cluster (Extended Data Fig. 5I), ranging from under 1% to over 40%; C3 and C4 were present in 6.4% and 9.4% of wild-type nuclei, respectively. Traces within each cluster share overall structural similarity rather than identical conformation, and individual traces probably vary in folding extent, including partially compacted or intermediate configurations. To confirm that the genotype-dependent enrichment patterns described above were not sampling artifacts, we performed three independent downsampling replicates (Extended Data Fig. 6a–i). These replicates reproduced the near-absence of C4 in condensin mutants (DS1 = 54, DS2 = 11, DS3 = 38 traces), supporting a genuine structural difference rather than a stochastic fluctuation in cluster assignment. This heterogeneity, together with the modest fraction of nuclei in any single state, may explain why these conformations are obscured in population-averaged maps and resolvable only through single-molecule clustering.

Across all three genotypes, the predominant configuration was cluster C1, a highly interactive domain spanning the entire region with intermediate folding and no clear boundaries (Fig. 4d), accounting for ~40% of traces per genotype. Subtle differences were evident: H3K9me mutants showed sharper proximity decline with genomic distance, consistent with a role in stabilizing long-range interactions, while dpy-28 mutants showed a checkered interaction pattern (also visible in compact domains like C3), weaker in wild type and smoother in H3K9me mutants. Together, these results reveal the genetic requirements of distinct domains at both large and finer scales.

To assess chromatin organization within elCID and TADL across conditions, we quantified pairwise inter-region distances across all loci within each domain on chromosome V (elCID, 18.2–19.6 Mb; TADL, 19.7–20.8 Mb) in wild-type and mutant embryos (Methods and Fig. 4e–h). The elCID region showed modest increases in median distances (0.62 µm vs 0.59 µm in wild type; Fig. 4e,f), indicating relative structural stability, whereas the TADL region displayed pronounced, reproducible decompaction (Fig. 4g) with significance maintained across nearly all iterations (Fig. 4h). These results indicate that TADL loci exhibit canonical, SMC-dependent TAD behavior, whereas elCID persists largely independently of condensin.

### Domain-like chromatin organization across multiple chromosomal regions

**Fig. 5:**
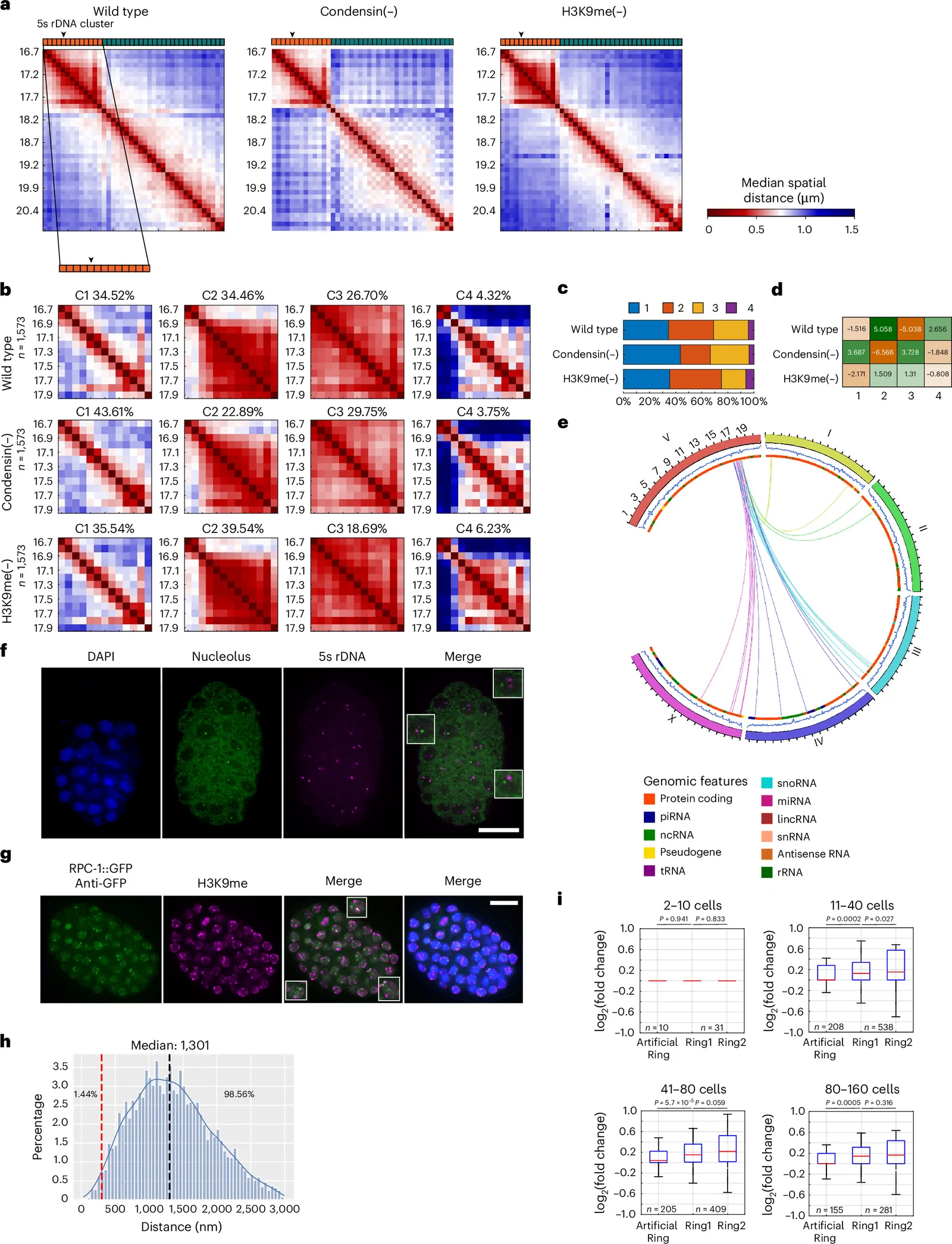
Chromatin organization of the 5SL elCID domain is preserved across genotypes. **a**, Population-averaged pairwise distance maps (μm) of the extended chromatin region, revealing the 5SL elCID domain (17.0–17.9 Mb) across wild-type, H3K9me and condensin (dpy-28) mutant embryos. **b**, Median pairwise distance matrices (μm) for clusters identified by unsupervised co-clustering of chromosome traces pooled across genotypes with matched age distribution. **c**, Distribution of cluster frequencies across genotypes. **d**, Heatmap of signed −log_10_(FDR) enrichment scores for each cluster, with green and orange indicating enrichment and depletion relative to wild type, respectively. **e**, Intrachromosomal Hi-C contact profiles showing interactions of the 5SL elCID domain with other genomic regions. **f**, Representative images showing the spatial relationship between the 5S rDNA locus and the nucleolus (FIB-1::GFP); representative of two independent experiments with similar results. **g**, Representative images of 5S rDNA loci with H3K9me3 immunostaining, indicating localization within heterochromatin; RPC-1 foci mark sites corresponding to 5S rDNA loci; representative of two independent experiments with similar results. Scale bar, 10 μm. **h**, Distribution of distances between the 5S rDNA locus and the nucleolus or RNA polymerase III (Pol III) foci, with 98.6% of measurements exceeding 300 nm. **i**, Spatial relationship between Pol III foci and H3K9me3-marked chromatin, showing reduced H3K9me3 within Pol III foci and enrichment in surrounding regions. *n* indicates the number of rings in each comparison. Statistical significance was determined using a two-tailed *t*-test. Source data

**Extended Data Fig. 7:**
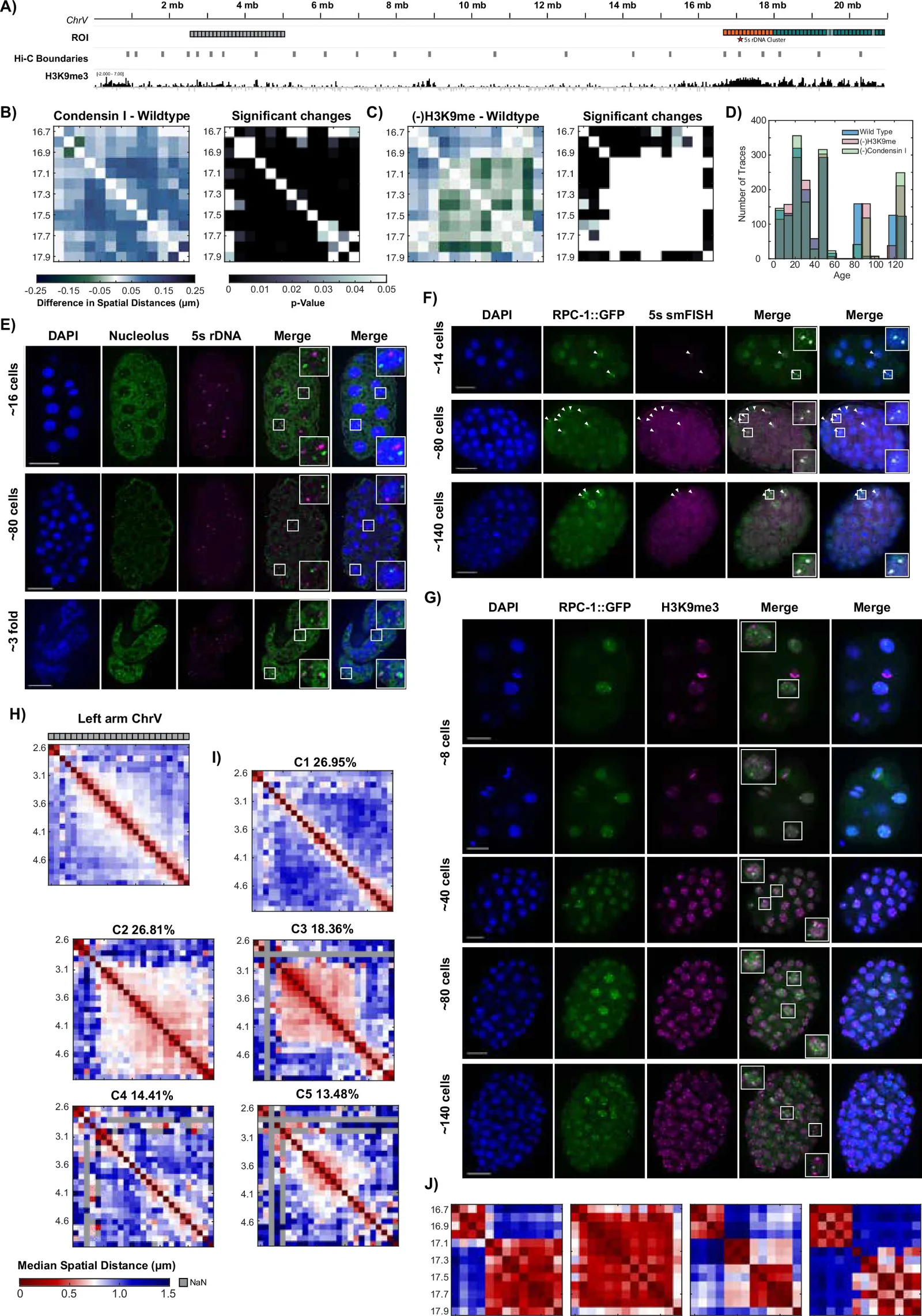
Quantitative analysis and additional chromatin tracing of the 5SL elCID domain. **A**) Schematic of the genomic regions analyzed in this study. The grey region corresponds to the left arm of chromosome V previously traced in Sawh et al. 2020, the orange region denotes the 5SL elCID domain, and the green region indicates the previously traced region shown in Figs. 1–4. **B,C**) Differences in pairwise distances (μm) between condensin (dpy-28) or H3K9me3 mutant and wild-type traces. Blue indicates increased distances in mutants, whereas green indicates increased distances in wild type. Corresponding significance maps are shown alongside. **D**) Age distribution of traces used for co-clustering in Fig. 5b. **E**) Representative images showing the spatial relationship between the 5S rDNA locus and the nucleolus (FIB-1::GFP) across developmental stages. **F**) Representative images showing the spatial relationship between the 5S rDNA locus and RNA polymerase III (RPC-1::GFP) foci across developmental stages. **G**) Representative images of 5S rDNA loci with H3K9me3 immunostaining across developmental stages, indicating localization within heterochromatin. RPC-1 foci mark sites corresponding to 5S rDNA loci. **H**) Population-averaged pairwise distance maps (μm) for additional heterochromatic regions (grey region). **I**) Clustering analysis of these regions reveals additional domain-like conformations not evident in population-averaged maps. **J**) Representative single-chromosome spatial distance maps (µm) showing domain-like structures in experimental traces.

To determine whether additional condensin I-independent domains exist, we extended chromatin tracing ~1.3 Mb upstream, uncovering a well-defined domain spanning 17.0–17.9 Mb, maintained in wild-type, H3K9me and condensin I mutant embryos. We refer to this region as the 5SL elCID domain, as it contains 5S rRNA and SL trans-spliced leader repeat genes. A sharp boundary separating the 5SL elCID from the adjacent elCID domain was preserved upon disruption of condensin or H3K9me3, with correspondingly infrequent interactions between the two (Fig. 5a and Extended Data Fig. 7a–c).

Unsupervised co-clustering of the 5SL elCID across genotypes, with normalized age distribution, identified discrete conformations detected in all genotypes but at differing frequencies (χ^2^_10_ = 541.95, *P* = 4.7 × 10^−^^110^; Cramer’s *V* = 0.23; Fig. 5b and Extended Data Fig. 7d). Cluster C2 was reduced in condensin mutants (22.9% vs 34.5% in wild type) and cluster C3 in H3K9me mutants (18.7% vs 26.7%; Fig. 5c,d), but no cluster was uniquely lost in either background, indicating that 5SL elCID architecture was largely preserved despite loss of condensin I or H3K9me3.

The 5S rRNA and SL1 loci are transcribed by RNA polymerase III and II, respectively. To assess whether the 5SL domain preferentially interacts with other regions, we analyzed intrachromosomal Hi-C contacts from a published dataset^31^. Contacts were observed with heterochromatin-rich regions on chromosomes X and III, but no enrichment for specific loci (Fig. 5e). Despite its role in ribosome biogenesis, the 5S rDNA locus did not associate with the nucleolus (median distance, 1,301 nm; 98.6% of measurements, >300 nm; FIB-1 marker; Fig. 5f,h and Extended Data Fig. 7e), indicating that the 5SL elCID is both spatially and genomically insulated. RNA FISH with immunostaining showed that most nuclei contained two distinct Pol III foci mapping to the 5S rRNA genes, which did not merge into one domain and did not always colocalize with H3K9me3 (Fig. 5f,g and Extended Data Fig. 7f,g). H3K9me3 was reduced within foci but enriched in surrounding chromatin, indicating that Pol III foci occupy transcriptionally permissive microenvironments adjacent to, but not embedded within, heterochromatin (Fig. 5i and Extended Data Fig. 7g).

Finally, we analyzed published chromatin tracing data at comparable resolution^33^, focusing on a 2 Mb region in another heterochromatic context (Extended Data Fig. 7h–j). This region lacked prominent domain structure at the population level (Extended Data Fig. 7h), but clustering revealed two clusters with clear boundaries, accounting for ~45% of traces, alongside highly extended conformations reflecting substantial cell-to-cell variability (C2–C3; Extended Data Fig. 7i,j). Together, these findings suggest that megabase-scale domain organization is a widespread feature of heterochromatin, with some regions resembling TADs and others elCIDs.

### Distinct genomic and chromatin features at the boundaries of TADL versus elCID domains

**Fig. 6:**
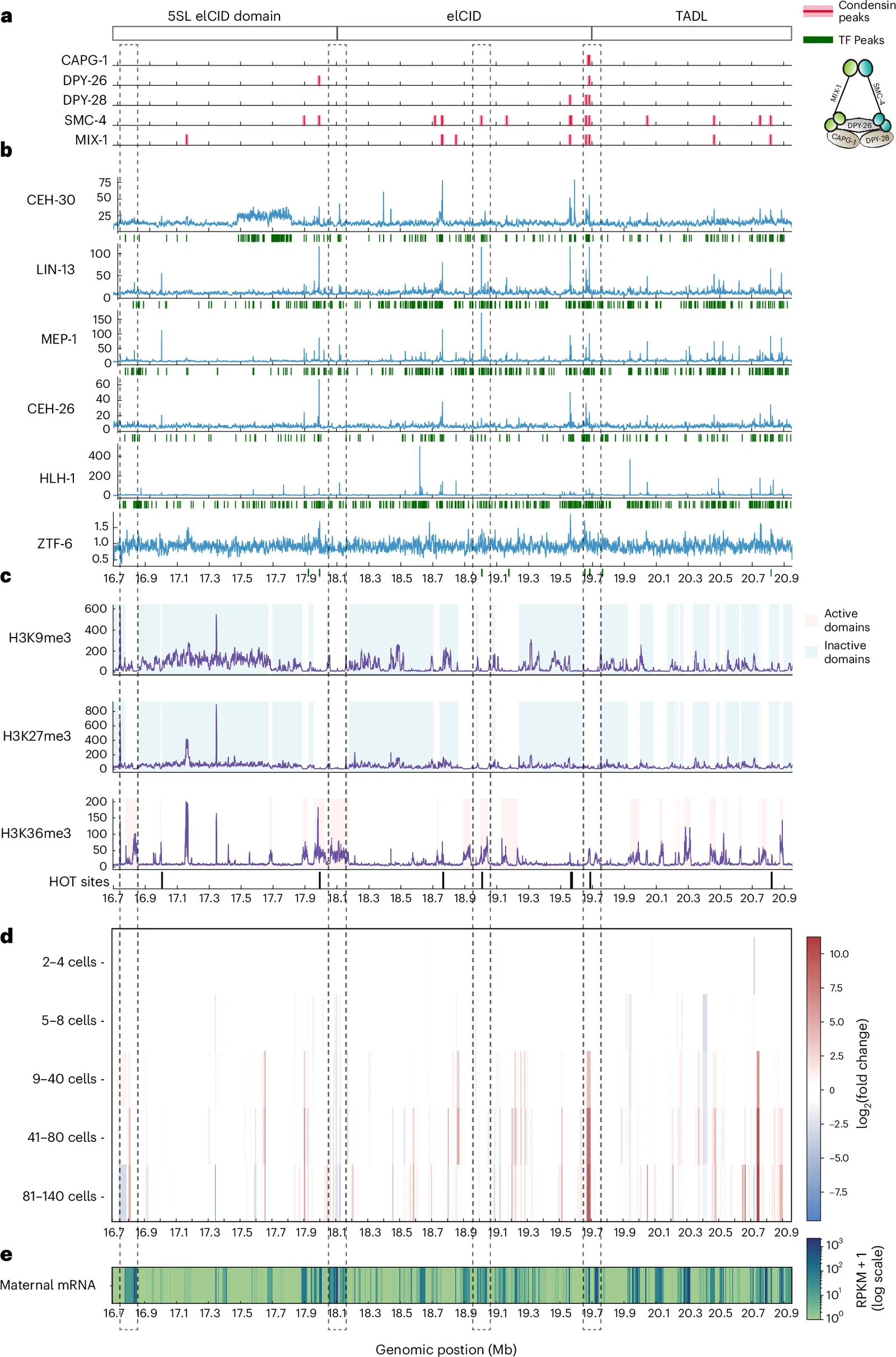
Chromatin features and transcriptional landscape across the 5SL elCID–TADL region. **a**, ChIP–seq peaks for condensin I subunits (CAPG-1, DPY-26 and DPY-28) across the analyzed chromosome V locus, previously identified^52^. A schematic model of the condensin I complex is shown on the right^41^. **b**, ChIP–seq signal for the top five transcription factors at condensin-bound sites in embryos. Signal intensity is shown in blue, and peak positions are indicated by green bars. **c**, ChIP–seq profiles for H3K9me3, H3K27me3 and H3K36me3 across the analyzed chrV locus^76^. Shaded regions indicate active domains (pink) and regulated domains (blue)^77^. **d**, The log_2_(fold change) of differentially expressed genes across the region at different developmental stages^54^. Gray boxes indicate boundaries identified by chromatin tracing and clustering. The dashed lines mark regions at 19.0 Mb with high boundary probability and at 16.8 Mb, identified as a boundary by visual inspection, as it lies at the edge of the traced region. **e**, Maternal mRNA levels before the 2-cell stage across the analyzed region^54^.

In vertebrate euchromatin, CTCF marks many TAD boundaries. *C.* *elegans* lacks an obvious CTCF ortholog, raising the question of what serves as a boundary element here. We analyzed published chromatin immunoprecipitation followed by sequencing (ChIP–seq) data for candidate factors^52^ and showed that TADL and elCID boundaries had different attributes. At the TADL boundary (~19.7 Mb), we observed peaks for all five subunits diagnostic of condensin I (Fig. 6a), raising the possibility that autosomal boundaries, like those on the X chromosome, are established by condensin I, analogous to the condensin I^DC^ complex^22,30^. This boundary was also marked by a HOT site, a break in H3K9me3 and H3K27me3 and peaks of DNA-binding proteins CEH-30, LIN-13, MEP-1 and CEH-26, previously linked to condensin binding^52^ (Fig. 6b,c), although only condensin I co-occupancy was unique to this position. A signature of zygotic transcription emerged at the 9–40-cell stage (Fig. 6d), after TADL establishment, suggesting zygotic transcription responds to, rather than induces, the boundary.

At the elCID boundary (18.1 Mb), peaks of CEH-30, LIN-13 and MEP-1 coincided with strong H3K36me3, depletion of H3K9me3 and H3K27me3 and robust maternal but minimal zygotic expression (Fig. 6b–e). A secondary boundary at ~19.0 Mb showed similar but more broadly distributed features, with less distinct transcription factor occupancy, and a boundary at ~16.8 Mb, marking the start of the 5SL elCID, showed the same pattern: reduced repressive marks, enriched H3K36me3, weak transcription factor occupancy and strong maternal expression. These attributes suggest distinct boundary elements for each domain type: condensin I for TADL, both here and on the X chromosome, whereas chromatin marks and gene activity may define elCID boundaries.

The three boundaries (18.1, 19.0 and 19.7 Mb) were enriched for repeats relative to the genome average, with region-specific enrichment for long terminal repeat elements and DNA transposons at 18.1 Mb and satellite, simple and rolling-circle repeats at 19.7 Mb (Supplementary Table 3). Given their proposed role in boundary formation^53^, we also examined C2H2 zinc finger motifs and found modest enrichment of ZTF-6 near the 19.0 Mb boundary, confirmed by ChIP–seq at 19.0 Mb and 19.7 Mb with additional peaks near 18.1 Mb (Supplementary Table 4). These data suggest that different domain types rely on distinct factors and chromatin attributes to form boundaries.

### Domain compaction and elCID, but not TADL domains, emerge progressively during development

To examine how heterochromatin domains arise during development, we analyzed chromosome structure across five embryonic stages: 2–4 cells (pre-zygotic genome activation (ZGA)), 5–8 cells (minor ZGA), 9–40 cells (gastrulation onset, first major ZGA) and 41–80 and 81–140 cells, coinciding with progressive H3K9 methylation accumulation^50,51^, using co-clustering and power-law scaling analysis to quantify compaction over time.

**Fig. 7:**
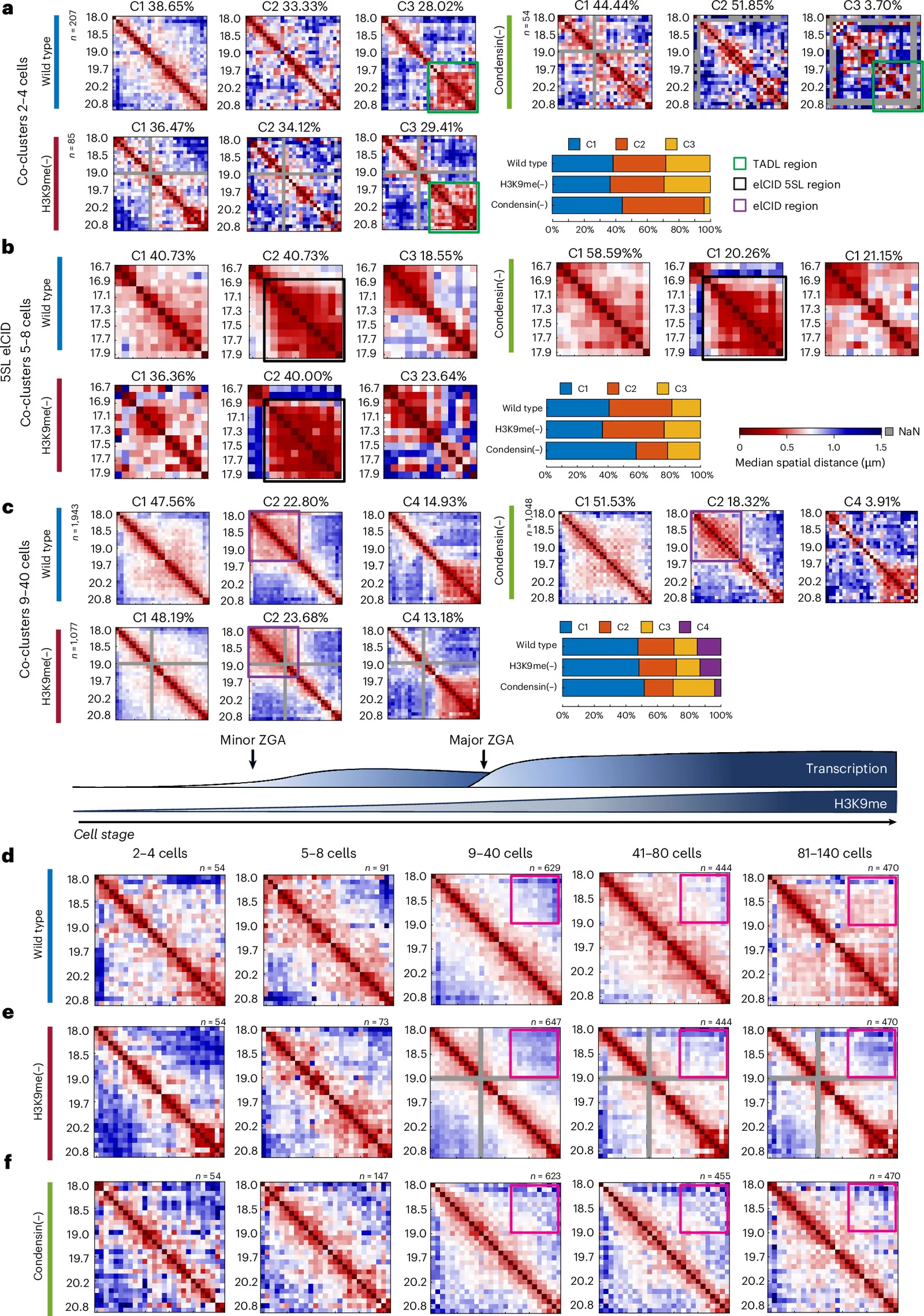
Onset of heterochromatin formation, chromatin compaction and higher-order domain organization during development. **a**, Median pairwise distance matrices (µm) for embryonic co-clusters at the 2–4-cell stage, separated by genotype. **b**, Median pairwise distance matrices (µm) for embryonic co-clusters at the 5–8-cell stage, for the 5SL elCID region. **c**, Median pairwise distance matrices (µm) for embryonic co-clusters at the 9–40-cell stage. In **a**–**c**, stacked bar charts show the fractional contribution of each genotype to each cluster; green boxes indicate the TADL domain, black boxes indicate the 5SL elCID and purple boxes indicate the elCID region. *n* indicates the number of traces. **d**–**f**, Average pairwise distance matrices across developmental age groups for wild type (**d**), H3K9me mutants (**e**) and condensin mutants (**f**). Magenta boxes highlight long-range interaction regions that change during development. NaN (gray) indicates missing values. *n* indicates the number of traces.

The TADL was detectable before ZGA in ~30% of wild-type and H3K9me mutant embryos (Fig. 7a). We analyzed published single-cell RNA-seq data^54^ at the same developmental stage and detected no transcriptional activity across the examined region, while during the minor ZGA, a few loci became weakly expressed in this region (Fig. 6d). These data indicate that the TADL domain emerges in the absence of transcription.

**Extended Data Fig. 8:**
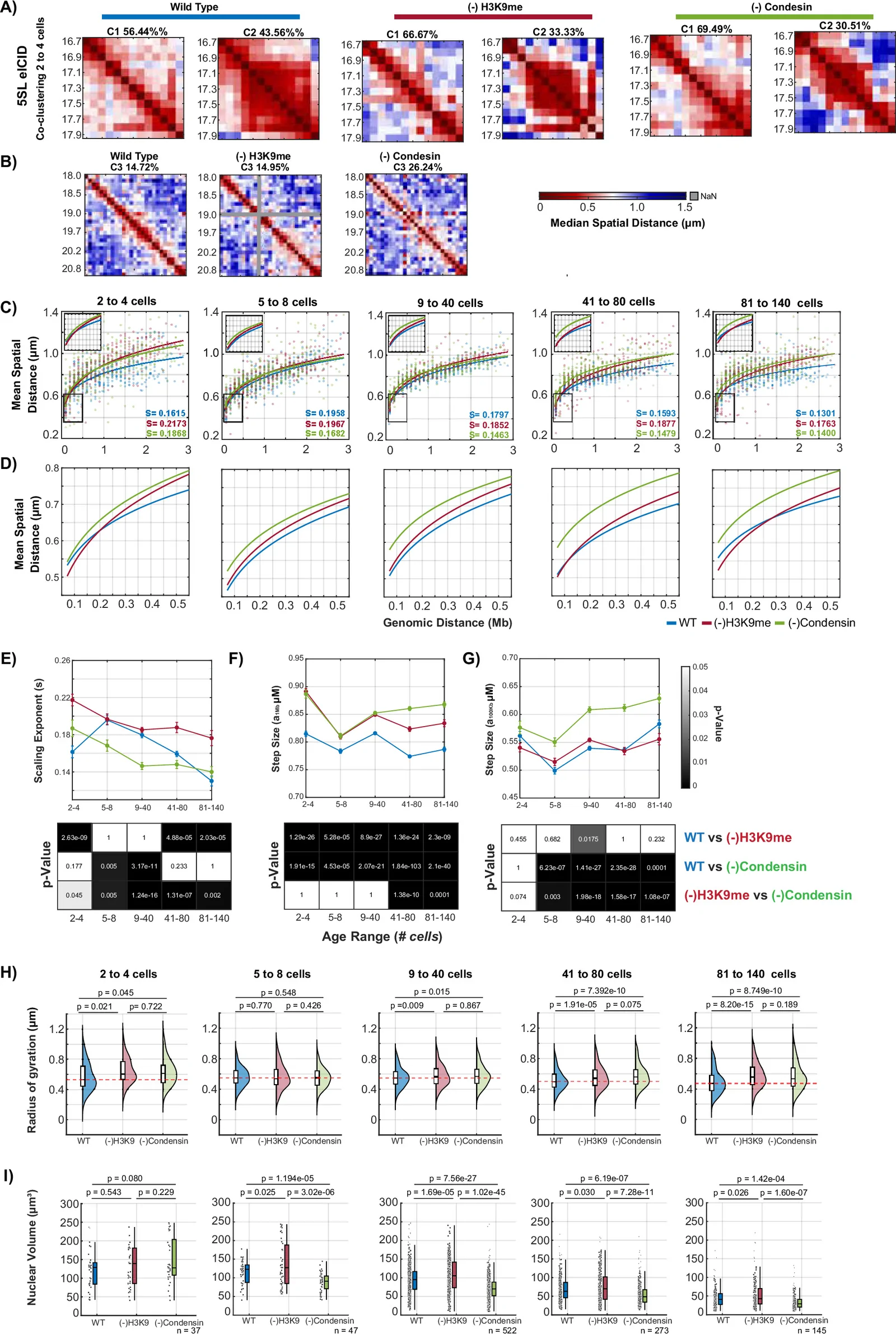
Developmental progression of chromatin structure across later embryonic stages. **A**) Median pairwise distance matrices (µm) for co-clusters from the 5SL elCID region at the 2–4 cell stage, separated by genotype. **B**) Median pairwise distance matrices (µm) for Cluster 3 from the co-clusters shown in Fig. 7c. **C**) Scaling of mean pairwise distance versus genomic distance for wild-type (blue), H3K9me(–) (red), and Condensin(–) (green) across five developmental stages. The black box highlights distances below 0.5 Mb, shown in detail in D. **D**) Zoomed-in view of the scaling of mean pairwise distance versus genomic distance below 500 kb for wild-type (blue), H3K9me(–) (red), and Condensin(–) (green) across the five developmental stages shown in Fig. 5e. **E**) Scaling exponent (s), and **F**–**G**) step sizes (a) at 1 Mb and 100 kb, estimated from log–log linear regression of spatial versus genomic distances. Error bars represent propagated standard errors. Pairwise Z-tests with Bonferroni correction were used to assess significance; corrected p-values are shown below each plot. **H**) Radius of gyration for wild-type (blue), H3K9me(–) (red), and Condensin(–) (green) across the five developmental stages. Statistical significance was determined using a two-tailed t-test. **I**) Nuclear volume across developmental stages and genotypes (µm^3^). Box plots indicate the median (center line) and 25th and 75th percentiles (box bounds); violin outlines show the full distribution of the data. n indicates the number of traces. Dashed red line indicates the wild-type median.

The condensin-independent elCID domains became visible at distinct stages. The 5SL elCID was detected at the minor ZGA (cluster C2, ~40% of wild-type traces; Fig. 7b) in all three genotypes, although it did not fully extend across the region in condensin or H3K9me mutants at earlier stages (Extended Data Fig. 8a,b), suggesting independence from transcription, absent in this region before the 9-cell stage (Fig. 6d). The second elCID emerged at the 9–40-cell stage across genotypes (Fig. 7c), becoming less frequent suggesting a transient structure; its higher transcriptional activity and emergence coinciding with the onset of transcription suggest transcription may contribute to its formation.

Compaction and compartment formation occurred in wild type but failed in both H3K9me and condensin I mutants (Fig. 7d–f), with long-range interactions visible within and between the second elCID and TADL domains in wild type (Fig. 7d, magenta box). To quantify folding, we analyzed the scaling of mean spatial distances with genomic distance (*R* = *a* *×* *N*^*s*^), where the exponent (*s*) reflects folding and the step size (*a*) reflects packing density. In wild type, scaling exponents declined from the 4-cell stage (*s* = 0.19 → 0.13; Extended Data Fig. 8c–g), consistent with progressive compaction. H3K9me mutants remained elevated throughout development, indicating persistent decompaction, while condensin mutants showed lower exponents than wild type early on (*s* = 0.16 vs 0.19) but converged to wild-type levels by the 80-cell stage. Per-trace 95% confidence intervals were relatively narrow, indicating consistent scaling despite moderate heterogeneity (Supplementary Table 5). The step sizes remained higher in both mutants at 1 Mb, reflecting expanded chromatin, and only condensin mutants showed elevated step sizes at 100 kb, pointing to a unique defect in local folding (Extended Data Fig. 8c,d). RoG measurements confirmed enlarged chromosome volumes in both mutants from the 9-cell stage onward (Extended Data Fig. 8h). Together, these results show that in the wild type, elCID and the TADL domain are compacted and appear to form a joint compartment over time (Fig. 7d).

Lastly, we tested whether these chromatin defects could be explained by nuclear size. H3K9me mutants maintained nuclear volumes comparable to wild type, indicating the chromatin changes are not caused by nuclear scaling (Extended Data Fig. 8i); dpy-28 embryos, by contrast, had significantly smaller nuclei despite increased chromatin volume, confirming these effects are not secondary to nuclear size.

### Single-cell chromatin domains persist despite loss of population TADLs and arise from cooperative long-range interactions

**Fig. 8:**
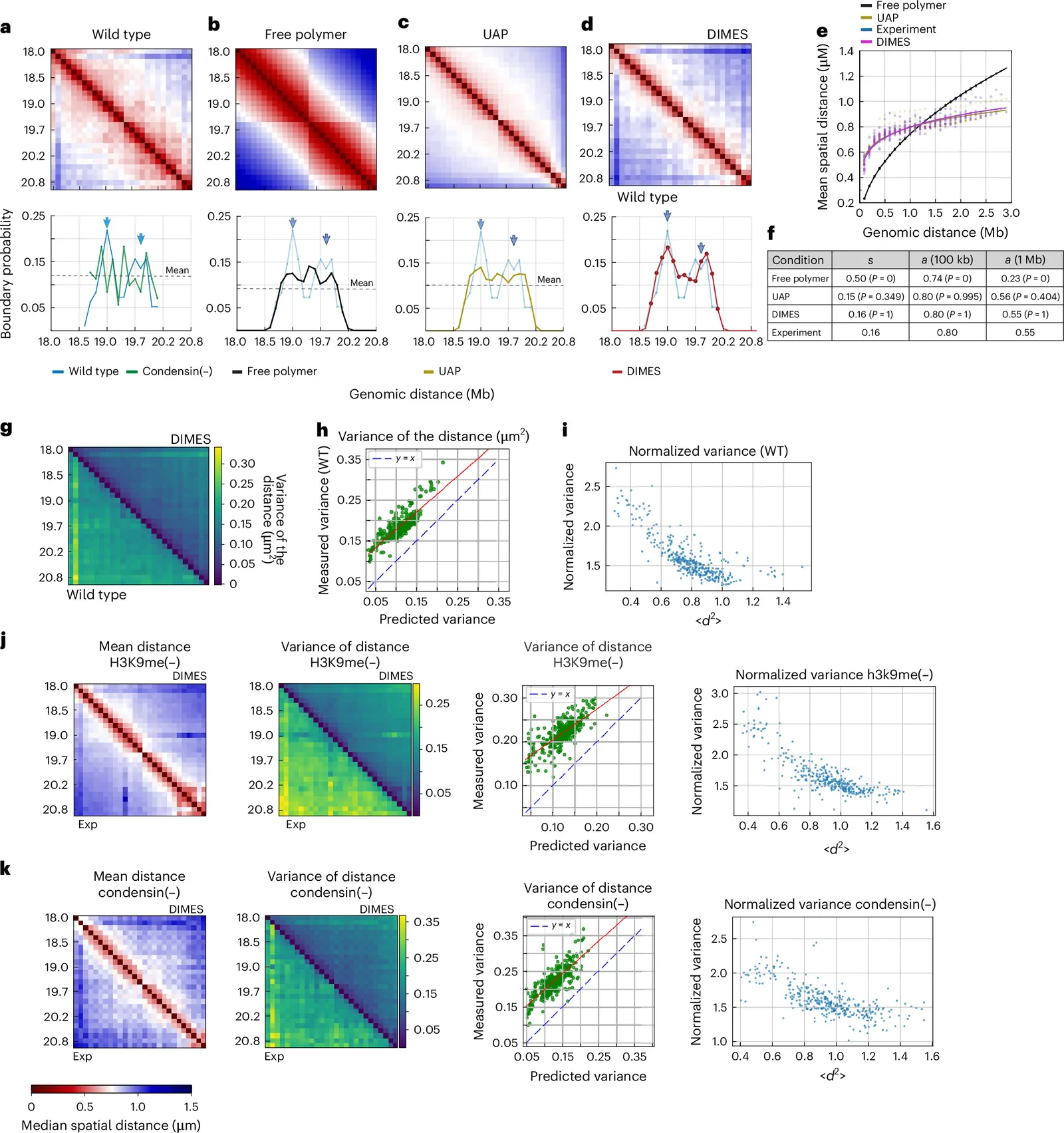
Chromatin variability and domain structure require structured interactions beyond generic polymer behavior. **a**–**c**, Top, median pairwise distance matrices; bottom, boundary probability profiles derived from single-chromosome traces. Boundary probability reflects the fraction of traces in which a genomic bin is classified as a boundary (see Methods) for the wild-type experiment (**a**), free polymer baseline (**b**) and UAP baseline (**c**). **d**, Top, mean pairwise distance matrices, with WT experiment (bottom left) and DIMES (top right); bottom, corresponding boundary probability profiles. **e**, Scaling relationships between genomic and spatial distance for experimental data, free polymer and UAP baselines. **f**, Summary of scaling exponents (*s*) and step sizes (*a*) at 100 kb and 1 Mb for each condition. Parameters were compared between datasets using two-tailed *z*-tests based on estimated values and their standard errors, with Bonferroni correction for multiple comparisons. **g**, Variance of pairwise distance matrices, with WT experiment (bottom left) and DIMES (top right). **h**, Scatter plot comparing variance of pairwise distances between experiment and DIMES. **i**, Scatter plot of normalized variance versus mean squared distance (*d*^2^) for wild type. **j**,**k**, Left to right, mean pairwise distance matrix, variance matrix, variance scatter plot and scatter plots of normalized variance versus mean squared distance for H3K9me(−) embryos (**j**) and condensin(−) embryos (**k**).

**Extended Data Fig. 9:**
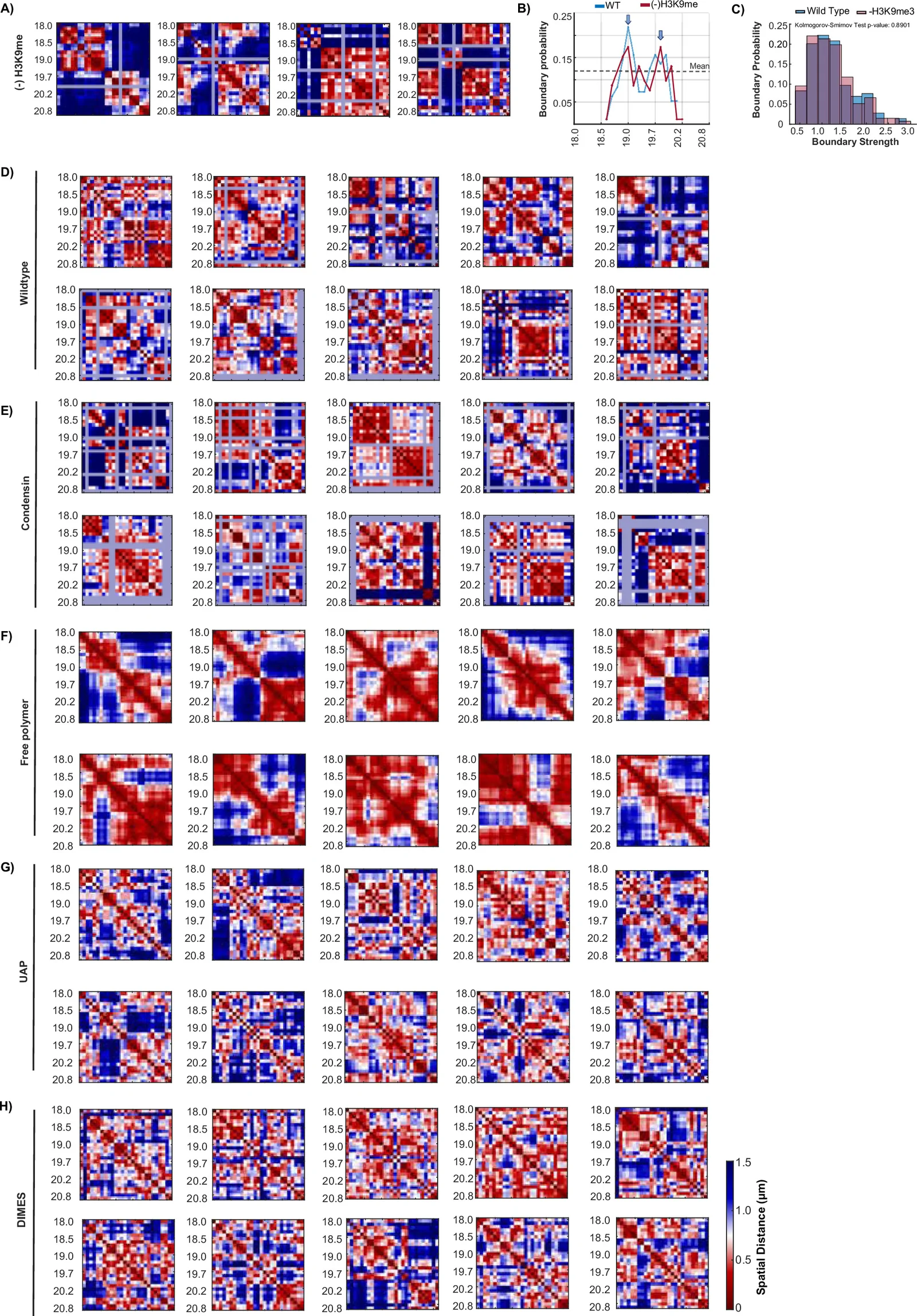
Representative single-chromosome spatial distance maps across experimental and simulated conditions. **A**) Representative single-chromosome spatial distance maps (µm) showing domain-like structures in H3K9me mutant experimental traces. **B**) Boundary probability profile for wild-type and H3K9me mutant embryos; blue arrows indicate regions of elevated boundary probability. **C**) Distribution of boundary strength in wild-type and H3K9me(−) embryos. Distributions were compared using a two-sample Kolmogorov–Smirnov (K–S) test. **D**–**H**) Randomly selected single-trace spatial distance matrices (μm) for wild-type (**D**), condensin(-) (**E**), free polymer simulations (**F**), UAP simulations (**G**), DIME (**H**). Each matrix represents a single chromosome trace, highlighting the variability in domain organization within and across conditions. NaN (gray) indicates regions with missing values.

A puzzling attribute of TADs in other animals is that structures similar to TADL domains persist in single-molecule traces of SMC mutants but vanish in population-averaged maps^47,55^. Similarly, single-molecule traces from condensin I and H3K9me mutant embryos retained domain-like structures (Extended Data Fig. 9a–e). We quantified boundary probabilities (Methods) and found elevated probabilities in wild type at 19.0 Mb (~20%) and 19.7–19.9 Mb (~15%), matching the boundaries identified by clustering and marking the elCID-to-TADL transition (Fig. 8a). These boundary probability percentages are similar to values reported for human cells^47,55^. In *dpy-28* mutants, boundary probabilities were evenly distributed across the region (Fig. 8a, green line), indicating increased variability in boundary positioning. In H3K9me mutant embryos, boundary positions remained centered near 19.0 Mb and 19.8 Mb, with slightly reduced probabilities, and overall boundary strength was unchanged (Kolmogorov–Smirnov test, *P* = 0.89; Extended Data Fig. 9b,c), suggesting a minor role for H3K9me in reinforcing boundaries. Together, these results show that chromatin domains persist at the single-cell level in the absence of condensin or H3K9me, like other animals^47,55,56^.

**Extended Data Fig. 10:**
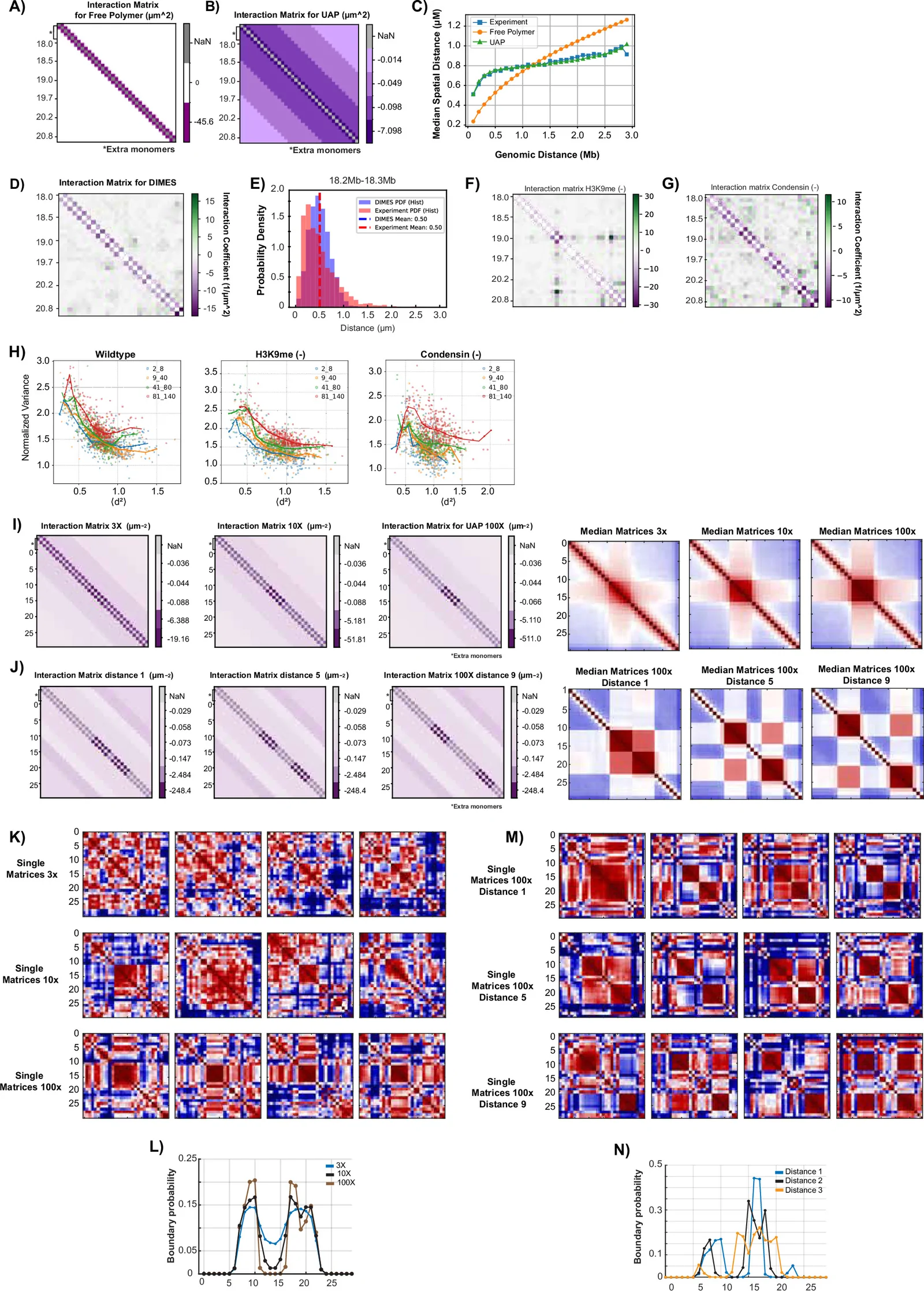
Comparison of chromatin structure between experimental data and polymer baselines. **A**-**B**) Interaction matrices used in the different models. **A**) Free polymer baseline; asterisks (*) indicate additional monomers included to minimize boundary effects. **B**) Uniformly attractive polymer (UAP) baseline. **C**) Scaling relationship between genomic distance and mean spatial distance for experimental data, free polymer, and UAP models. **D**) DIMES model for wild-type embryos. **E**) Distribution of pairwise distances for an example locus pair (~2.9 Mb separation), comparing experiment (red) and DIMES (blue). **F**-**G**) Interaction matrices for DIMES models in mutant embryos. **F**) H3K9me(−). **G**) Condensin(−). **H**) Normalized variance plotted against mean squared distance 〈d^2^〉 for wild-type, H3K9me(−), and condensin(−) embryos. Points represent locus pairs and are color-coded by developmental stage (2–8, 9–40, 41–80, 81–140 cells per embryo). Solid lines indicate smoothed trends. **I**) Interaction matrices for models with enhanced intra-domain interactions (3×, 10×, 100×), shown alongside corresponding median distance matrices from simulations. **J**) Interaction matrices for the 100× model with two domains separated by 1, 5, or 9 regions, shown alongside corresponding median pairwise distance matrices (µm). **K**) Representative single-molecule distance matrices for single-domain models in (I). **L**) Boundary probability profiles for simulations in (I). **M**) Representative single-molecule distance matrices for two-domain models in (J). **N**) Boundary probability profiles for simulations in (J).

To distinguish biologically relevant variability from intrinsic polymer fluctuations, we applied a statistical framework modeling chromatin as a Gaussian process defined by a pairwise interaction matrix^57,58^. This framework provides a statistical null baseline for features, such as boundary probability and excess variability, that cannot be explained by generic polymer behavior alone. We generated three baselines of increasing complexity (10,000 conformations each; Fig. 8b–d and Extended Data Figs. 9f–h and 10a–d). Even the simplest case—a free polymer with nearest-neighbor interactions—produced apparent boundaries and insulation, calibrated to the experimental RoG, but failed to capture distance scaling and boundary probabilities (Fig. 8b,e,f and Extended Data Fig. 10c). Adding uniformly attractive long-range interactions improved agreement with wild-type scaling and compaction (root mean squared error (RMSE) reduced from 0.186 to 0.057) but still failed to reproduce boundary probabilities (Fig. 8c and Extended Data Fig. 10b,c), indicating that structured, locus-specific interactions are required.

As a third case, we used Distance Matrix to Ensemble of Structures (DIMES)^58^ to generate ensembles matching experimental mean pairwise distances (RMSE = 0 by design; Fig. 8d and Extended Data Fig. 10d). DIMES recapitulated boundary positions but with a shallower profile and significantly underestimated variance, particularly for proximal pairs (Fig. 8d,g–i and Extended Data Fig. 10e), demonstrating that average structure and polymer fluctuations alone cannot explain the heterogeneity observed in vivo. To determine whether specific biological perturbations alter this excess variability, we applied DIMES to mutant conditions (Extended Data Fig. 10f,g). In H3K9me mutant embryos, normalized variance remained comparable to wild type (Fig. 8j), indicating that H3K9 methylation does not substantially contribute to chromatin heterogeneity. In condensin mutant embryos, normalized variance was reduced, particularly at short genomic distances (Fig. 8k), identifying condensin I as a major source of structured chromatin variability. Normalized variance increased during development in all genotypes (Extended Data Fig. 10h) despite differences in compaction, suggesting that chromatin heterogeneity grows progressively and is only revealed by single-molecule measurements.

Introducing enhanced intra-domain interactions showed that contacts must be very strong to produce robust single-molecule domains with distinct boundary signals (Extended Data Fig. 10i–n). Simulating domain pairs at varying separations revealed that closely spaced domains cooperatively reinforce boundaries (Extended Data Fig. 10j,m,n), explaining why elCID boundary probability is reduced upon condensin loss (Fig. 8a): although elCID itself is condensin-independent, the neighboring condensin-dependent TADL domain normally reinforces its boundary.

## Discussion

This study identifies megabase-scale domains in *C.* *elegans* heterochromatin, with distinct genetic requirements that define different categories. TADL domains are highly folded, with boundary probabilities comparable to mammalian cells^47,55,56^, and require condensin I. elCID domains resemble TADLs but are largely independent of condensin I or H3K9me. Although they depend on distinct factors for biogenesis, both domain types converge to promote compartment-like heterochromatin in older embryos.

The elCID and TADL domains differed in boundary composition: the left TADL boundary was marked by condensin I (the right coincides with the chromosome end), whereas elCID boundaries were associated with active histone marks. Both have precedent: condensin I^DC^ is required for TAD boundary formation on the X chromosome^22,31^, while active genes and histone marks have been implicated in domain formation in *C.* *elegans* and *Drosophila*^32,59,60^. A surprising feature of the elCID boundaries is that they correlate with maternal rather than ongoing zygotic gene expression, consistent with the maternal inheritance of H3K36me3 (ref. ^61^). This raises the possibility that elCID boundaries rely on maternal transcriptional activity.

TADL domains were detectable before ZGA, indicating they initiate independently of transcription. Earlier studies in other organisms reported conflicting relationships between TADs and ZGA^62–66^. Our findings suggest that these discrepancies may partly reflect population averaging, which can mask structures present in only a subset of chromosomes. In *C.* *elegans*, TADL represented a substantial but still minority fraction of traces before ZGA (~28%). By contrast, the second elCID domain appeared coincident with zygotic transcription, suggesting that this domain class may rely on transcriptional activity.

Previous studies concluded that *C.* *elegans* lacks TADs, based on the absence of a CTCF ortholog and the failure to detect sharp boundaries at the population level^23,67^. Our data suggest instead that this absence reflects conformational variability rather than a genuine lack of domains. It remains to be tested whether this reinterpretation extends to euchromatic regions or other chromosomes.

Boundary formation in other systems typically involves cooperation among multiple insulator proteins, often zinc finger-containing factors^53,68–73^. LIN-13, observed at the TADL boundary, co-occupies the genome with condensin I (ref. ^52^), and is also a binding partner of HPL-2/HP1 (refs. ^74,75^), raising the possibility that LIN-13 functions as both a boundary factor and heterochromatin organizer. Our motif analysis found a modest enrichment of a single factor, ZTF-6, near the elCID boundary. Whether any of these factors directly contribute to boundary formation remains to be determined.

One elCID domain encompassed the 5S and SL leader repeat locus. At the sequence level, these repeats are flanked by H3K9me3-marked heterochromatin, potentially analogous to the heterochromatin surrounding the nucleolus. The 5SL elCID differs from the nucleolus, however, in that the two loci remain spatially separated within nuclei. An intriguing possibility is that the 5S locus is packaged within a domain, analogous to other ribosomal genes.

Aside from elCID and TADL, most traces displayed less defined structures, consistent with reports that TADs account for only a small fraction of chromosome conformations in mammalian cells in vivo (≤3%)^21,37^. Such configurations are poorly captured by Hi-C but accessible through single-molecule imaging. Our statistical polymer baselines show that generic fluctuations can produce apparent boundaries but fail to account for measured boundary probabilities and excess variance. This discrepancy was genotype-dependent, with condensin loss reducing heterogeneity, unlike H3K9me loss, identifying condensin-mediated interactions as a major source of structured variability.

Our tracing focused on heterochromatic regions of chromosome V, providing a foundation for future genome-wide comparisons. However, a limitation of the study is the 100 kb probe spacing, which resolves megabase-scale architecture but not the finer features that coexist within these domains. Our statistical polymer framework offers a non-mechanistic baseline for detecting structural variability, and our TADL terminology similarly captures structural and genetic dependence on condensin I, but future studies are needed to model chromosome organization and to uncover their functions.

## Methods

### Worm strains

*C.* *elegans* strains used in this study included the N2 Bristol strain (wild type), GW638 *met-2(n4256) set-25(n5021)* III and TY4381 *dpy-28(s939)/qC1 [dpy-19(e1259) glp-1(q339) qIs26]* III. All strains were cultured at 20 °C on nematode growth medium plates seeded with OP50.

### Genomic data

#### Hi-C data

Hi-C data for *C.* *elegans* embryos (GSE206065)^31^ were used to generate contact matrices at 100 kb resolution, normalized using the iterative correction and eigenvector decomposition method; regions excluded from tracing were similarly omitted from Hi-C plots. Pearson correlation confirmed strong agreement between averaged tracing distances and normalized Hi-C contact frequencies (Extended Data Fig. 1a). Hi-C boundary information for Fig. 1a was taken from prior work (GSM1556154)^22^.

To evaluate cohesin and condensin perturbation, Hi-C datasets from GSE199723 (ref. ^23^) were binned at 100 kb resolution to match the tracing scale. Distance-dependent contact probability and log_2_(fold change) relative to wild type were computed for genomic separations of ~100 kb–2 Mb on chromosome V and the X chromosome.

#### Circos plot

To visualize interchromosomal contacts for the region of interest (ROI) on chromosome V, we generated circular plots using the pyCircos Python library. Hi-C balanced contact matrices from GEO accession GSE168803 (ref. ^31^) were loaded at 10 kb resolution with the Cooler Python library. Interchromosomal contacts were identified and statistically evaluated using FitHiC2 (ref. ^78^), which estimates expected contact probabilities and assigns significance (*q* values) to each interaction. Significant interactions were defined as those with *q* < 10⁻^3^ and were retained for downstream visualization. Analyses were restricted to the ROI on chromosome V. Autosomes and the X chromosome were included in the circular layout, each represented as a distinct segment. Significant interchromosomal interactions were displayed as curved links colored by the target chromosome. Insulation scores were computed from the same Hi-C data and shown as an additional track together with genomic features annotated from the WormBase WBPS18 canonical gene set (PRJNA13758) to provide contextual information for the observed contacts.

#### Single-cell RNA-seq data analysis

Raw single-cell RNA-seq counts (GSE83523)^54^ were aggregated into pseudobulk samples by developmental range (0–1, 2–4, 5–8, 9–40, 41–80 and 81–140 cells) and replicate, matching the ranges used for chromatin tracing. Differential expression was assessed in DESeq2, using the 0–1-cell stage as reference to minimize maternal transcript influence; log_2_(fold changes) were shrunken with lfcShrink^79^, and likelihood ratio tests identified genes with significant expression changes across stages. Resulting log_2_(fold changes), normalized expression values and adjusted *P* values were used for downstream analyses.

#### CHIP–seq data analysis

ChIP–seq data for H3K9me3 (GSE2272039)^76^ were downloaded and visualized using Integrative Genomics Viewer (IGV). To characterize chromatin state across the chromosome V ROI, histone ChIP–seq bedGraph tracks for H3K9me3 and H3K27me3 (modENCODE_5163) and H3K36me3 (modENCODE_5165) were obtained from WormBase and smoothed using a 2 kb rolling window before visualization. Chromatin domain annotations were incorporated from a previous publication^77^. Active and regulated domains were displayed as semi-transparent genomic spans over regions enriched for H3K36me3 and H3K9me3/H3K27me3, respectively.

Transcription factors enriched at condensin I-IDC binding sites in early embryos were selected based on the prior analysis^52^. The five most enriched transcription factors were CEH-30, LIN-13, MEP-1, CEH-26 and HLH-1. Processed ChIP–seq signal and peak tracks for these factors were downloaded from WormBase, corresponding to the following GEO datasets: CEH-30 (GSE25808), LIN-13 (GSE25804), MEP-1 (GSE25800), CEH-26 (GSE37798) and HLH-1 (GSE25784). MACS-called peak files for condensin subunits CAPG-1, DPY-26 (kleisin), DPY-28 (hCAPD2), DPY-27 (SMC4) and MIX-1 (SMC2) were downloaded from GEO (GSE45678) and used for visualization^52^. In addition, ZTF-6 ChIP–seq signal (fold change over control) was obtained from GSE257069 and used for plotting. Given that chromatin tracing was performed at 100 kb resolution, whereas the ChIP–seq datasets have kilobase-scale resolution (2 kb), these comparisons allow assessment of relative enrichment within traced regions.

#### Repeat and zinc finger motif analysis

Repeat annotations for chromosome V (UCSC RepeatMasker, ce11, Repbase^80^) were used to calculate per-1 kb-bin repeat density (fraction of bases overlapping annotated repeats) for each repeat class and combined. Rolling mean densities were computed over 150 kb windows; densities within ±75 kb of each ROI center (19.0 Mb, 19.75 Mb) were compared against background density estimated from 10,000 randomly sampled genomic positions.

For motif analysis, ±75 kb windows around each ROI were subdivided into 2 kb segments and scanned for 18 C_2_H_2_ zinc finger motifs (JASPAR Core) using the MEME Suite, against 450 random 2 kb background regions. The ZTF-6 motif was further scanned genome-wide across chromosome V using FIMO.

### Proteomics sample preparation and LC–MS/MS analysis

Embryos were dissected as for tracing experiments, flash-frozen and lysed (5% SDS, 10 mM TCEP, 0.1 M TEAB) by sonication (PIXUL, Active Motif), heated (95 °C, 10 min) and alkylated (20 mM iodoacetamide). Proteins were digested with S-Trap micro columns (Protifi) using sequencing-grade trypsin (1:25 w/w, Promega; 1 h, 47 °C), and eluted peptides were dried under vacuum.

Peptides were resuspended in 0.1% formic acid/0.02% DDM and analyzed by LC–MS/MS on a timsTOF Ultra 2 (Bruker, CaptiveSpray source) coupled to a Vanquish Neo UHPLC (Thermo), separated on a C18 column (100 µm × 30 cm, 1.5 µm ReproSil Saphir; 60 °C, 0.4 µl min^−1^) over a 2–95% acetonitrile gradient (~35 min). The mass spectrometer ran in dia-PASEF mode (100–1,700 *m*/*z*; eight scans × three ion-mobility windows, 400–1,000 *m*/*z*, 25 Da isolation; 1,600 V capillary voltage, 200 °C dry temperature), with collision energy ramped from 59 eV to 20 eV across the ion-mobility range.

Raw data were processed in Spectronaut (v.20.3, directDIA) against the *C.* *elegans* UniProt proteome (26,696 sequences) plus contaminants. Fragment-group intensities were analyzed in ProteoFlux v.1.8.0 (log-transformed, median-normalized, missing values imputed with lc_conmed), and differential abundance was tested using limma with Benjamini–Hochberg correction (Supplementary Table 1).

### Chromatin tracing

Tracing was conducted with probe sets at 100 kb resolution, using chromatin tracing as previously described^33^^,34^.

#### Sample preparation

Gravid young adult worms were dissected in double-distilled water to isolate embryos. The embryos were then transferred onto round cover slips pre-coated with poly-L-lysine for better adhesion. Embryos were fixed for 5 min in a solution of 1% paraformaldehyde (PFA) and 0.05% Triton X-100. The slides were placed on dry ice and freeze-cracked^35^.

For *dpy-28(s939)*, homozygous adults were identified by selecting worms with a wild-type, non-roller phenotype and confirming the absence of GFP signal. Embryos were collected from these verified homozygous mutant mothers, thus ensuring the absence of detectable maternal contribution (Extended Data Fig. 2a)^40^. The *dpy-28(s939)* allele has been previously characterized as a strong loss-of-function mutation that impairs condensin I activity^40,42^, although residual condensin activity may be partially retained.

#### Primary probe design

Probe design and synthesis methods were as previously described^35^. DNA sequence was extracted from the *C.* *elegans* genome assembly (ce11). Primary probes, each 102 nt in length, target consecutive 100 kb regions covering the distal right arm of chromosome V. Each primary probe sequence contains four parts: 5′ PCR-priming sequence (20 nt), genome homologous sequence (42 nt), readout sequence (20 nt) and 3′ PCR-priming sequence (20 nt) from prior work^81^. The OligoArray 2.1 (ref. ^82^) parameters used were: targeting region length, 42 nt; melting temperature, 78–100 °C; no internal secondary structure with melting temperature greater than 76 °C; no cross-hybridization with melting temperature greater than 76 °C. GC content 30–90% and sequences do not contain consecutive repeats of seven or more identical nucleotides. OligoArray v.2.1 was run on sciCORE for high-performance computing at the University of Basel. The 42 nt homologous sequences were further blasted by NCBI BLAST+ v.2.9.0 for specificity over the *C.* *elegans* genome.

#### Hybridization protocol

After freeze-crack fixation, samples were rinsed in PBS, washed three times in PBS/0.5% Triton X-100 and treated with RNase A (0.05 mg ml^−1^, 37 °C, 30 min) to digest RNA. Samples were then pre-hybridized (2× SSC, 10% dextran sulfate, 0.1% Tween-20, 50% formamide; 47 °C, 1 h). Primary probes, diluted 1:100 in hybridization buffer, were added and incubated for 10 min, followed by denaturation on a hot metal surface (85 °C, 10 min) and hybridization in a humid chamber for ≥16 h at 47 °C. Post-hybridization washes consisted of 1 h in 2× SSC/50% formamide, followed by two 30 min washes in 2× SSC and two in 0.5× SSC, all at 47 °C. Secondary probes (8 nM in 2× SSC/25% ethylene carbonate) were hybridized for 30 min, washed and imaged (561 nm or 647 nm). Across sequential rounds, displacement oligonucleotides removed bound secondary probes while new secondary probes for subsequent regions were introduced concurrently^33,36^.

#### Microscope imaging pipeline

Imaging used a widefield microscope (Ti2-Crest, Nikon) with a custom microfluidics system for secondary probe delivery^36,43^. Embryos were stained with DAPI, and fiducial beads (488 nm) were added for alignment. Primary probe and DAPI signals were captured per field of view, followed by primary probe photobleaching and sequential secondary probe imaging rounds. Tetraspeck beads were imaged each round for channel alignment. Average localization precision was ~50 nm^33,34^.

#### Imaging analysis

After tracing, the acquired images underwent foci fitting as previously described^33,34^. For image segmentation and chromatin tracing, MATLAB was used, implementing modified methods outlined in prior studies^33,36^. Initially, DAPI fluorescence was used to identify nuclei within each embryo. Background signals were subtracted, and noise was removed. The images were then converted to binary format, distance-transformed and processed using watershed segmentation, resulting in volumetric masks that delineated individual nuclei. Chromosome territories were segmented in a similar fashion, with the nuclear mask excluding signals outside the nuclei. This process produced a volumetric representation in which each volume corresponded to a distinct chromosome. Chromatin tracing was subsequently executed within these defined volumes using the nearest-neighbor approach as previously described^33^.

Median distance matrices used traces with ≥7 detected regions, averaged across replicates (which showed strong correlation). Regions 19.4, 19.5 and 20.6 Mb were excluded owing to undetectable hybridization signal; in H3K9me(−) datasets, region 19.0 Mb showed reduced coverage from a hybridization artifact and is shown as NaN where median distance could not be computed. After filtering, 165 wild-type (4,022 nuclei), 89 H3K9me(−) (3,547 nuclei) and 154 condensin(−) (5,715 nuclei) embryos were analyzed.

Detection efficiency was quantified from a binary detection matrix, which denotes successful detection of a locus in a chromosome trace. Average probe recovery was defined as the mean fraction of traces in which each locus was detected. Trace completeness was defined as the fraction of loci detected per trace, and the median trace completeness was reported for each dataset. After exclusion of low-quality loci, average probe recovery was 53.7% for wild type, 55.1% for H3K9me(−) and 44.9% for condensin(−). Median trace completeness was 51.9%, 55.6% and 40.7% for wild type, H3K9me(−) and condensin(−), respectively.

### Chromosome conformation analysis

#### Pearson correlation between replicates

For each genotype, a Pearson correlation coefficient was calculated to assess the mean pairwise spatial distance consistency between regions and across replicates. The MATLAB function corrplot was used for these analyses.

#### Visualization of individual traces

To visualize individual genomic region traces in three dimensions, we used the scatter3 function in MATLAB. Each trace was represented in a 3D space, with the *X*, *Y* and *Z* parameters corresponding to the coordinates of each point in the trace.

#### Insulation analysis

Insulation scores were calculated following previous work^22^, with modifications for tracing data. Mean distances were first normalized for genomic distance—accounting for non-consecutive hybridization regions—by fitting the data to a power-law function reflecting the expected relationship between spatial proximity and genomic distance^34^. A 200 kb sliding window was applied along the interaction matrix diagonal to average interactions across bins, with the mean signal assigned to the corresponding tracing region; edge bins were excluded, as no data were available for the first and last regions owing to insulation box constraints.

Scores were normalized per chromosome as the log_2_ ratio of each bin’s score to the chromosome-wide mean, allowing comparison of local insulation relative to overall organization. Local minima (‘valleys’), representing high insulation, were identified with a delta vector (the difference between mean insulation values 100 kb upstream and downstream of each bin), with valleys detected at zero-crossings. Boundary strength was quantified as the difference in delta values between the local maximum (left) and minimum (right), following prior work^22^; bins with boundary strength of ≥0.1 were classified as boundaries.

#### Boundary probability

Traces with ≥25 regions were used to generate a mean distance matrix, with linear interpolation across single missing points. Insulation analysis (300 kb insulation box, 200 kb delta box; boundary strength of ≥0.5) was applied per trace, and boundary probability was calculated across all traces, following previous publications^47,55^. Boundary strength and probability distributions were compared between genotypes using the two-sample Kolmogorov–Smirnov test.

#### Clustering

Clustering was conducted as previously described^33,36^. Pairwise distance vectors from each trace were assembled into a matrix; regions 2, 11, 17, 18 and 29 were excluded genome-wide owing to low detection. Matrices were normalized (LogNormalize, ScaleData; in Seurat) before dimensionality reduction to remove global compaction differences while preserving relative folding topology.

Clustering used Louvain community detection (in Seurat). Resolution was selected by systematically sweeping the Louvain resolution parameter and evaluating clustering quality using graph modularity and subsampling stability (adjusted Rand index); both metrics exhibited a broad optimum across intermediate resolutions (~0.8–1.2), with higher resolutions primarily subdividing clusters with similar average structures or generating small fragmentary clusters. A conservative resolution of 0.8 was therefore selected for wild-type analyses, preserving major structural states while avoiding over-partitioning and enabling comparison across datasets. Robustness was confirmed on two independent random 50% subsamples of wild-type traces, without replacement, clustered at resolution 1.0 (Extended Data Fig. 1e). Clusters within each dataset were ordered by boundary strength; resolution-sweep statistics are reported in Supplementary Table 2.

#### Median matrix comparison

Matrices from different genotypes were subtracted from one another. To test for significance, we performed a one-tailed Welch’s *t*-test on all pairwise distance measurements for each interaction. Unless otherwise noted, all mutant comparisons were performed relative to the wild-type dataset shown in Fig. 1.

#### Downsampling the chromosome between genotypes

To compare traces across genotypes (wild-type, H3K9me mutant, condensin mutant) without confounding by trace count or age distribution, chromosome ages were binned into five-age intervals, retaining only bins common to all three conditions. For each shared bin, traces were randomly downsampled to match the minimum count across genotypes (deterministic where the limiting genotype had all available traces); conclusions were unchanged across independent resampling replicates.

#### Iterative downsampling and Wilcoxon testing of spatial distances

To assess differences in spatial distance distributions between conditions, we performed repeated random downsampling followed by statistical testing using chromosome traces derived from the age-matched downsampling described above. Distances were computed across all loci within the specified elCID or TADL domains without restricting traces based on cluster assignment. For each genotype, distances were randomly subsampled (*n* = 1,000 per iteration), and the procedure was repeated across 100 independent iterations. In each iteration, we computed the median pairwise distance for loci within the TADL or elCID domains and evaluated pairwise differences between datasets using the Wilcoxon rank-sum test.

For each comparison, we quantified the fraction of iterations yielding *P* < 0.05 (reported as significance), which reflects the robustness of the observed differences across random subsets of the data.

#### RoG

The RoG was calculated for chromosome traces containing a minimum of 25 regions, as previously described^33,47^, with genotype differences assessed by *t*-test.

#### Co-clustering and chi-squared test for co-clusters

Age-matched, downsampled traces from N2, condensin(−) and H3K9me(−) embryos were pooled and clustered jointly (see ‘Clustering’) to identify structural states shared across genotypes as well as genotype-specific states. Resolution was selected by sweeping values and evaluating modularity and subsampling stability (adjusted Rand index); clustering quality plateaued at a resolution of 0.5, as higher resolutions mainly fragmented similar structural classes without improving modularity or stability, so 0.5 was used for downstream analyses. Traces were then separated by genotype to visualize cluster distributions. For the 5SL elCID domain, traces were filtered to ≥5 detected regions and clustered at a resolution of 0.35, yielding four clusters.

Differences in cluster distribution across genotypes were assessed using Pearson’s chi-squared test of independence on genotype–cluster contingency tables (chi-squared statistic was computed following a previous publication^36^; all expected cell counts >5), with Cramér’s *V* quantifying effect size. Standardized residuals identified genotype–cluster combinations contributing most to the global association, indicating both direction and magnitude of enrichment or depletion; per-cluster enrichment relative to wild type (N2) was further tested by a 2 × 2 chi-squared test with Benjamini–Hochberg correction. Enrichment scores for visualization were calculated as signed −log_10_(FDR), signed by the standardized residual direction (positive, enrichment; negative, depletion relative to N2).

Nuclear fractions were calculated by first assigning each traced allele to its parent nucleus, identified by mapping the allele’s coordinates onto the DAPI-based nuclear segmentation mask. Traces were grouped by nucleus identity (sample, field of view and nucleus label); nuclei with more than two associated territories were excluded as probable segmentation artifacts. The fraction of nuclei in each state, and the fraction showing mixed conformations, was calculated separately for each genotype.

#### Power-law fitting

To compare scaling parameters across datasets, we performed log–log linear regressions (log(*D*) = log(*a*) + *s* × log(*G*), where *D* is mean spatial distance and *G* is genomic distance) to estimate the scaling exponent *s* and step size *a* at 1 Mb and 100 kb. Standard errors from regression confidence intervals were propagated to step-size uncertainties (100 kb value computed as *a* × (0.1)^*s*^), and pairwise *z*-tests compared parameters between datasets using their estimated values and standard errors, with Bonferroni correction for multiple comparisons (significance threshold of *P* < 0.05 after correction).

To assess variability across traces, scaling exponents were also estimated independently per trace by log–log regression, with mean and 95% confidence intervals calculated across traces per condition (two-sided *t*-distribution; Supplementary Table 5).

### Microscopy experiments

#### Antibody staining

Samples were fixed with 1% PFA (5 min), followed by cold methanol (5 min, –20 °C), then washed three times in PBS/0.5% Triton X-100 (10 min each) and twice in PBS, all at 22 °C room temperature. Samples were blocked in 4% BSA/PBS (30 min, room temperature), then incubated with ChromoTek GFP-Booster (gb2AF488) or anti-H3K9me3 (ab8898), diluted 1:500 in blocking buffer, for 1 h at room temperature. After three PBS washes (5–10 min each), secondary antibody was applied for 1 h at room temperature. A second fixation (1% PFA, 5 min) stabilized the signal before downstream DNA or RNA FISH.

#### FLAP-Y smFISH probe preparation and hybridization

Equimolar 5S rDNA probes (5′-ACAACATCCAGGATTCCCAGGCCGTC-3′ and 5′-AACTTGCCAGATCGGACGGGATGGCGT-3′) were pooled and diluted fivefold in IDTE buffer (pH 8.0; final concentration, 0.833 µM per probe). For pre-hybridization, a 20 µl reaction (4 µl primary probe pool, 1 µl secondary FLAP-Y probe, 2 µl 10× NEBuffer 3, 13 µl nuclease-free water) was thermocycled (85 °C, 3 min; 65 °C, 3 min; 25 °C, 5 min) and stored at 4 °C (ref. ^83^).

For hybridization, 100 µl hybridization solution (10% dextran sulfate in 1:1:8 formamide:20× SSC:water) was applied and incubated (37 °C, 1 h), then replaced with 50 µl fresh solution containing 1 µl pre-hybridized probes (37 °C, 4 h, dark). Samples were washed twice in wash buffer (1:1:8 formamide:20× SSC:water), then incubated in 100 µl wash buffer (37 °C, 1 h, dark), washed twice more and mounted in Vectashield with DAPI (H-1200, Vector Laboratories).

#### Imaging acquisition

Imaging used a spinning-disk confocal system (Olympus IX83, Yokogawa CSU-W1, 50 µm disk; cellSens software), with a Hamamatsu ORCA-Fusion sCMOS camera and ×60 oil-immersion objective (NA, 1.5).

#### smFISH count and distance pipeline

Embryos and nuclei were segmented using Cellpose-SAM, applied to a Gaussian-blurred FISH channel (embryos) and raw DAPI images (nuclei). FISH dot detection used an in-house Python pipeline: images were deconvolved with a microscope-specific point spread function (pycudadecon), then filtered with a Difference of Gaussians filter to enhance FISH puncta, and dots were segmented by dataset-specific intensity thresholding of the Difference of Gaussians-filtered images. Semantic masks were converted to instance masks (SciPy or CuPy label function), and small artifacts (<100 pixels) were removed. Dots were assigned to nuclei by mask overlap, with centroids computed from voxel-based masks; dot count was the total detections per nucleus or embryo. For distance measurements, dots from paired FISH channels were matched per nucleus by nearest-neighbor centroid distance to avoid cross-nucleus assignments.

#### H3K9me3 enrichment surrounding Pol III foci

To analyze H3K9 methylation enrichment around Pol III foci, we segmented foci as for FISH detection and generated two concentric 200 nm rings extending outward from each focus, yielding three zones: the core focus and two peripheral rings. A low-threshold mask restricted the H3K9me3 signal to regions of detectable fluorescence, avoiding skew from its concentration in discrete foci. Median H3K9me3 intensity in each zone was normalized to mean nuclear intensity and zone area, and fold enrichment was calculated by comparing each ring’s normalized intensity to that of the core focus, with significance assessed using a Wilcoxon signed-rank test.

### Statistical polymer baselines

#### Multivariate normal sampling

To provide a minimal statistical baseline to distinguish biologically regulated from intrinsic polymer fluctuations, we used a Gaussian framework in which polymer configurations are drawn from a multivariate normal (MVN) distribution. Pairwise interactions between loci are encoded in an interaction matrix, which specifies the covariance matrix that fully constrains the MVN distribution. Specifically, for a polymer with *n* monomers, configurations are sampled from the following distribution:

$$P({x})={(2\pi )}^{-3n/2}{\det ({\Sigma })}^{-1/2}\exp \left(-\frac{1}{2}{x}^{T}{{\Sigma}}^{-1}x\right)$$

In the interaction matrix, attractive interactions were assigned negative values, while repulsive interactions were positive. The interaction matrix was constructed as a Kirchhoff matrix by setting each diagonal entry such that its corresponding row sums to zero. Given that the Kirchhoff interaction matrix has zero determinant owing to translational symmetry, we removed the last row and column and fixed the corresponding monomer at the origin (0,0,0). This eliminates the global translational degree of freedom and ensures the matrix can be used for MVN sampling. To generate three-dimensional structures, the interaction matrix was replicated across each of the three diagonal blocks of the full precision matrix. Chromatin conformations were sampled from an MVN distribution using PyTorch’s torch.distributions.MultivariateNormal, with the interaction matrix provided as the precision matrix.

In both the free polymer and uniformly attractive polymer (UAP) baselines, five additional monomers were appended to the 5′ end to eliminate the terminus effect, as only the 3′ end corresponds to the actual chromatin terminus. These extra monomers were excluded from the final output to ensure alignment with the experimental dataset.

### Parameters

#### Free polymer

In the free polymer baseline, we only included uniform nearest-neighbor interactions. The value of the interaction coefficient was set to 45.6 µm^−2^ so that the mean RoG of the free polymer configurations matched the wild-type experimental dataset at 0.55 µm. Pairs with longer distances were set to zero interaction strength (Extended Data Fig. 9).

#### UAP

In the UAP baseline, we set interaction strength based on the distance between the pair of regions. The values were adjusted to fit the folding relationship (Extended Data Fig. 9). Four different values were used: 7.098 µm^−2^ for nearest neighbors (0.1 Mb), 0.098 µm^−2^ for pairs 0.2–0.9 Mb apart, 0.049 µm^−2^ for pairs 1–1.9 Mb apart and 0.0147 µm^−2^ for pairs ≥2 Mb apart (Extended Data Fig. 9). We note that these coefficients are not uniquely determined, as different sets of values might similarly reproduce the observed distance relationship.

#### Domain-forming polymers

To investigate whether localized interaction strengthening can generate domain-like structures, we constructed domain-forming models as perturbations of the UAP baseline. In single-domain models, interaction strengths within a contiguous region of five consecutive nearest-neighbor pairs were increased by 3×, 10× or 100× relative to baseline, then re-optimized using the UAP fitting procedure to preserve global scaling while maintaining the relative enhancement within the domain. Double-domain models introduced two such 100×-enhanced regions, separated by one, five or nine regions, with interactions similarly re-optimized to preserve global scaling. All matrices were constructed in Kirchhoff form and used as precision matrices for MVN sampling; the full set is provided in the associated GitHub repository (https://github.com/dcpb0/Chromosome-domains.git).

#### DIMES

We inferred effective interaction matrices from experimental data using the DIMES framework according to a previous publication^58^, which implements a maximum entropy approach to determine the interaction matrix that reproduces the observed mean pairwise distances under a Gaussian polymer model.

The mean distance matrix derived from experimental data was used as input (input-type: dmap; input-format: text). The resulting connectivity matrix represents an effective interaction matrix whose inverse corresponds to the covariance structure of an equivalent MVN polymer model.

To ensure compatibility with our MVN sampling framework, the inferred matrix was symmetrized to correct for numerical asymmetries. Given that DIMES defines interactions with an opposite sign convention, we used the negative of the connectivity matrix as the precision matrix Λ for MVN sampling.

As with other models, the translational degree of freedom was removed by excluding the last row and column, and the corresponding monomer was fixed at the origin (0,0,0). The resulting matrix was then used to generate polymer conformations by MVN sampling.

#### Normalized variance

When the coordinates follow a MVN distribution, the pairwise distance follows a Maxwell–Boltzmann distribution, and therefore the ratio of variance and mean squared distance $\frac{\mathrm{var}}{\left\langle {d}^{2}\right\rangle }$ has a fixed ratio of $\frac{3\pi -8}{3\pi }$. The normalized variance is calculated as $D=\frac{3\pi }{3\pi -8}\frac{\mathrm{var}}{\left\langle {d}^{2}\right\rangle }$.

#### Mean distance matrix comparison

We quantified the mean distance differences between experimental data and different models using the RMSE over unique monomer pairs after removing unavailable regions. The RMSE is defined as:

$${RMSE}=\sqrt{\frac{1}{M}\sum _{i < j}{\left({D}_{{ij}}^{\left(1\right)}-{D}_{{ij}}^{(2)}\right)}^{2}}$$

where ${D}^{(1)}$ and ${D}^{(2)}$ are the mean distance matrices being compared, and M is the number of included monomer pairs. This metric provides a global measure of discrepancy in pairwise distances without requiring structural alignment.

#### Scaling analysis of spatial versus genomic distance

To quantify the scaling relationship between genomic distance and mean spatial distance, both quantities were log transformed, converting the power-law relationship into a linear form. Specifically, we modeled:

$$\log \left\langle {d}_{{ij}}\right\rangle ={\beta }_{0}+{\beta }_{1}\log \left(\left|i-j\right|\right)+{\beta }_{2}G+{\beta }_{3}\log \left(\left|i-j\right|\right)\times G+\epsilon$$

where $\left\langle {d}_{{ij}}\right\rangle$ denotes the mean spatial distance between loci *i* and j, $\left|i-j\right|$ represents their genomic separation and G is a binary variable indicating the dataset (experimental data versus model). In this formulation, ${\beta }_{0}$ and ${\beta }_{1}$ correspond to the intercept and scaling exponent for the reference dataset (G=0), while ${\beta }_{2}$ and ${\beta }_{3}$ quantify differences in intercept and scaling exponent between datasets.

To assess differences in scaling behavior, we tested the significance of ${\beta }_{3}$ (difference in scaling exponent). In addition, differences in mean spatial distance at specific genomic scales were evaluated by testing linear combinations of coefficients, ${\beta }_{2}G+{\beta }_{3}\log (x)$, at x=1 and x=10, corresponding to genomic distances of 100 kb and 1 Mb, respectively. Statistical significance of these terms was used to quantify differences between experimental data and models.

### Reporting summary

Further information on research design is available in the Nature Portfolio Reporting Summary linked to this article.

## Online content

Any methods, additional references, Nature Portfolio reporting summaries, source data, extended data, supplementary information, acknowledgements, peer review information; details of author contributions and competing interests; and statements of data and code availability are available at https://doi.org/10.1038/s41594-026-01895-4.

## Supplementary information

Reporting Summary

Supplementary Table 1 Supplementary Table 1. Protein abundance results from label-free LC–MS/MS quantification.

Supplementary Table 2 Supplementary Table 2. Resolution-sweep statistics for co-clustering analysis.

Supplementary Table 3 Supplementary Table 3. Repeat element density and enrichment at the 18.1, 19.0, and 19.7 Mb chromatin domain boundaries.

Supplementary Table 4 Supplementary Table 4. C2H2 zinc finger transcription factor motif enrichment at chromatin domain boundaries.

Supplementary Table 5 Supplementary Table 5. Mean scaling exponents (s) and 95% confidence intervals.

## Source data

Source Data Fig. 5 Quantification data from Fig. 5.

## Data Availability

All data generated in this study have been deposited at https://github.com/dcpb0/Chromosome-domains.git. Original microscopy images are available from the authors upon request owing to dataset size, except for images related to Fig. 7, which are deposited at Zenodo (https://zenodo.org/records/21773207)^84^. Proteomic data have been deposited at ProteomeXchange (https://www.proteomexchange.org) through MassIVE (https://massive.ucsd.edu) under identifiers MSV000102679 and PXD081978. Source data are provided with this paper.

## Code availability

Code used in this study was adapted from Sawh et al., 2020 (ref. ^33^), and/or developed by the authors, and is available at https://github.com/dcpb0/Chromosome-domains.git.
