# Supplementary material for: Single-molecule chromatin tracing reveals a diversity of megabase heterochromatin domains
Source: Nat Struct Mol Biol. 2026 Oct 2;33(10):1454–68. doi: 10.1038/s41594-026-01895-4 (PMC13672103; doi:10.1038/s41594-026-01895-4)
Supplement: Supplementary file 1 [file 41594_2026_1895_MOESM1_ESM.pdf]

nature portfolio

Corresponding author(s): Susan E Mango

Last updated by author(s): Aug 4, 2026

Reporting Summary

Nature Portfolio wishes to improve the reproducibility of the work that we publish. This form provides structure for consistency and transparency in reporting. For further information on Nature Portfolio policies, see our Editorial Policies and the Editorial Policy Checklist.

Statistics

For all statistical analyses, confirm that the following items are present in the figure legend, table legend, main text, or Methods section.

n/a	Confirmed	
☐	☒	The exact sample size (n) for each experimental group/condition, given as a discrete number and unit of measurement
☐	☒	A statement on whether measurements were taken from distinct samples or whether the same sample was measured repeatedly
☐	☒	The statistical test(s) used AND whether they are one- or two-sided<br>*Only common tests should be described solely by name; describe more complex techniques in the Methods section.*
☐	☒	A description of all covariates tested
☐	☒	A description of any assumptions or corrections, such as tests of normality and adjustment for multiple comparisons
☐	☒	A full description of the statistical parameters including central tendency (e.g. means) or other basic estimates (e.g. regression coefficient) AND variation (e.g. standard deviation) or associated estimates of uncertainty (e.g. confidence intervals)
☐	☒	For null hypothesis testing, the test statistic (e.g. F, t, r) with confidence intervals, effect sizes, degrees of freedom and P value noted<br>*Give P values as exact values whenever suitable.*
☒	☐	For Bayesian analysis, information on the choice of priors and Markov chain Monte Carlo settings
☒	☐	For hierarchical and complex designs, identification of the appropriate level for tests and full reporting of outcomes
☐	☒	Estimates of effect sizes (e.g. Cohen's d, Pearson's r), indicating how they were calculated

Our web collection on statistics for biologists contains articles on many of the points above.

Software and code

Policy information about availability of computer code

Data collection	Microscopy images for chromatin tracing were acquired using a Nikon Ti2-Crest widefield microscope integrated with a custom microfluidics system. smFISH and antibody images were acquired on an Olympus IX83 inverted microscope, with image acquisition and system control managed via cellSens software
Data analysis	All data analyses were performed using custom scripts developed in MATLAB and Python or adapted from Sawh et al., 2020. These scripts are included in the deposited code repository https://github.com/dcpb0/Chromosome-domains.git.

For manuscripts utilizing custom algorithms or software that are central to the research but not yet described in published literature, software must be made available to editors and reviewers. We strongly encourage code deposition in a community repository (e.g. GitHub). See the Nature Portfolio guidelines for submitting code & software for further information.

Data

Policy information about availability of data

All manuscripts must include a data availability statement. This statement should provide the following information, where applicable:

- Accession codes, unique identifiers, or web links for publicly available datasets
- A description of any restrictions on data availability
- For clinical datasets or third party data, please ensure that the statement adheres to our policy

The code used in this study was adapted from Sawh et al., 2020, and/or developed by the authors. All data and code generated in this study have been deposited in https://github.com/dcpb0/Chromosome-domains.git. Original microscopy images are available from the authors upon request due to the large size of the dataset.

Research involving human participants, their data, or biological material

Policy information about studies with human participants or human data. See also policy information about sex, gender (identity/presentation), and sexual orientation and race, ethnicity and racism.

Reporting on sex and gender	*Use the terms sex (biological attribute) and gender (shaped by social and cultural circumstances) carefully in order to avoid confusing both terms. Indicate if findings apply to only one sex or gender; describe whether sex and gender were considered in study design; whether sex and/or gender was determined based on self-reporting or assigned and methods used. Provide in the source data disaggregated sex and gender data, where this information has been collected, and if consent has been obtained for sharing of individual-level data; provide overall numbers in this Reporting Summary. Please state if this information has not been collected. Report sex- and gender-based analyses where performed, justify reasons for lack of sex- and gender-based analysis.*
Reporting on race, ethnicity, or other socially relevant groupings	*Please specify the socially constructed or socially relevant categorization variable(s) used in your manuscript and explain why they were used. Please note that such variables should not be used as proxies for other socially constructed/relevant variables (for example, race or ethnicity should not be used as a proxy for socioeconomic status). Provide clear definitions of the relevant terms used, how they were provided (by the participants/respondents, the researchers, or third parties), and the method(s) used to classify people into the different categories (e.g. self-report, census or administrative data, social media data, etc.) Please provide details about how you controlled for confounding variables in your analyses.*
Population characteristics	*Describe the covariate-relevant population characteristics of the human research participants (e.g. age, genotypic information, past and current diagnosis and treatment categories). If you filled out the behavioural & social sciences study design questions and have nothing to add here, write "See above."*
Recruitment	*Describe how participants were recruited. Outline any potential self-selection bias or other biases that may be present and how these are likely to impact results.*
Ethics oversight	*Identify the organization(s) that approved the study protocol.*

Note that full information on the approval of the study protocol must also be provided in the manuscript.

Field-specific reporting

Please select the one below that is the best fit for your research. If you are not sure, read the appropriate sections before making your selection.

☒ Life sciences ☐ Behavioural & social sciences ☐ Ecological, evolutionary & environmental sciences

For a reference copy of the document with all sections, see nature.com/documents/nr-reporting-summary-flat.pdf

Life sciences study design

All studies must disclose on these points even when the disclosure is negative.

Sample size	Experiments were performed on multiple embryos per genotype. Only chromosome traces with a minimum of seven genomic regions detected were included in the analyses. The number of traces used for each genotype is indicated in the figures (wild-type = 5,169; dpy-28 = 3,432; met-2,set-25 = 4,078). For down-sampled comparisons across genotypes, chromosomes were randomly selected from age-matched bins to ensure equal representation across conditions (n=1688). For the 5SL domain traces with minimum five detected region were use and were randomly selected from age-matched bins to ensure equal representation across conditions (n=1573)
Data exclusions	Regions 19.4, 19.5, and 20.6 were excluded from analyses due to absence of detectable hybridization signals. In H3K9me(-) datasets, region 19.0 was excluded because of an artifact caused by low hybridization efficiency. Regions 2, 11, 17, 18, and 29, which showed low detection in one or more datasets, were excluded from clustering analyses in all genotypes. No other data points were excluded. All procedures are detailed in the Methods section.
Replication	All experiments, including chromosome tracing, hybridization, and imaging, were independently replicated across multiple embryos, with three biological replicates for wild-type and Condensin(-) and two biological replicates for H3K9me(-) mutants.

Randomization	For comparisons across genotypes, chromosomes were randomly selected from each age bin to match the minimum number of traces across conditions, mitigating potential confounding from uneven sampling. Otherwise, embryos were processed under identical conditions, and no additional randomization was applied.
Blinding	Blinding was not performed during sample preparation or imaging, as these procedures were standardized and automated where possible. Data analysis, including foci identification, segmentation, and tracing, was performed using automated MATLAB pipelines, reducing potential bias in outcome measurements.

Reporting for specific materials, systems and methods

We require information from authors about some types of materials, experimental systems and methods used in many studies. Here, indicate whether each material, system or method listed is relevant to your study. If you are not sure if a list item applies to your research, read the appropriate section before selecting a response.

Materials & experimental systems

n/a	Involved in the study	
☐	☒	Antibodies
☒	☐	Eukaryotic cell lines
☒	☐	Palaeontology and archaeology
☐	☒	Animals and other organisms
☒	☐	Clinical data
☒	☐	Dual use research of concern
☒	☐	Plants

Methods

n/a	Involved in the study	
☒	☐	ChIP-seq
☒	☐	Flow cytometry
☒	☐	MRI-based neuroimaging

Antibodies

Antibodies used	ChromoTek GFP-Booster (gb2AF488) or Anti-H3K9me3 (ab8898)
Validation	Antibodies were obtained from commercial sources (ChromoTek GFP-Booster gb2AF488; Abcam Anti-H3K9me3 ab8898) and used according to the manufacturers' recommendations. Both antibodies have been validated by the manufacturers for the reported applications. The Anti-H3K9me3 antibody (ab8898) has been widely used and validated in published studies. We have also validated these antibodies against negative controls.

Animals and other research organisms

Policy information about studies involving animals; ARRIVE guidelines recommended for reporting animal research, and Sex and Gender in Research

Laboratory animals	C. elegans strains were used in this study (N2 Bristol strain (wild-type), GW638 met-2(n4256) set-25(n5021) III and TY4381 dpy-28(s939)/qC1 [dpy-19(e1259) glp-1(q339) qIs26] III).
Wild animals	*Provide details on animals observed in or captured in the field; report species and age where possible. Describe how animals were caught and transported and what happened to captive animals after the study (if killed, explain why and describe method; if released, say where and when) OR state that the study did not involve wild animals.*
Reporting on sex	*Indicate if findings apply to only one sex; describe whether sex was considered in study design, methods used for assigning sex. Provide data disaggregated for sex where this information has been collected in the source data as appropriate; provide overall numbers in this Reporting Summary. Please state if this information has not been collected. Report sex-based analyses where performed, justify reasons for lack of sex-based analysis.*
Field-collected samples	*For laboratory work with field-collected samples, describe all relevant parameters such as housing, maintenance, temperature, photoperiod and end-of-experiment protocol OR state that the study did not involve samples collected from the field.*
Ethics oversight	*Identify the organization(s) that approved or provided guidance on the study protocol, OR state that no ethical approval or guidance was required and explain why not.*

Note that full information on the approval of the study protocol must also be provided in the manuscript.

Plants

Seed stocks	*Report on the source of all seed stocks or other plant material used. If applicable, state the seed stock centre and catalogue number. If plant specimens were collected from the field, describe the collection location, date and sampling procedures.*
Novel plant genotypes	*Describe the methods by which all novel plant genotypes were produced. This includes those generated by transgenic approaches, gene editing, chemical/radiation-based mutagenesis and hybridization. For transgenic lines, describe the transformation method, the number of independent lines analyzed and the generation upon which experiments were performed. For gene-edited lines, describe the editor used, the endogenous sequence targeted for editing, the targeting guide RNA sequence (if applicable) and how the editor was applied.*
Authentication	*Describe any authentication procedures for each seed stock used or novel genotype generated. Describe any experiments used to assess the effect of a mutation and, where applicable, how potential secondary effects (e.g. second site T-DNA insertions, mosiacism, off-target gene editing) were examined.*
